# Diagnostic Performance of Artificial Intelligence–Based Methods for Tuberculosis Detection: Systematic Review

**DOI:** 10.2196/69068

**Published:** 2025-03-07

**Authors:** Seng Hansun, Ahmadreza Argha, Ivan Bakhshayeshi, Arya Wicaksana, Hamid Alinejad-Rokny, Greg J Fox, Siaw-Teng Liaw, Branko G Celler, Guy B Marks

**Affiliations:** 1 School of Clinical Medicine, South West Sydney UNSW Medicine & Health UNSW Sydney Sydney Australia; 2 Woolcock Vietnam Research Group Woolcock Institute of Medical Research Sydney Australia; 3 Graduate School of Biomedical Engineering UNSW Sydney Sydney Australia; 4 Tyree Institute of Health Engineering UNSW Sydney Sydney Australia; 5 Ageing Future Institute UNSW Sydney Sydney Australia; 6 BioMedical Machine Learning Lab Graduate School of Biomedical Engineering UNSW Sydney Sydney Australia; 7 Informatics Department Universitas Multimedia Nusantara Tangerang Indonesia; 8 NHMRC Clinical Trials Centre Faculty of Medicine and Health University of Sydney Sydney Australia; 9 School of Population Health and School of Clinical Medicine UNSW Sydney Sydney Australia; 10 Biomedical Systems Research Laboratory School of Electrical Engineering and Telecommunications UNSW Sydney Sydney Australia; 11 Burnet Institute Melbourne Australia

**Keywords:** AI, artificial intelligence, deep learning, diagnostic performance, machine learning, PRISMA, Preferred Reporting Items for Systematic Reviews and Meta-Analysis, QUADAS-2, Quality Assessment of Diagnostic Accuracy Studies version 2, systematic literature review, tuberculosis detection

## Abstract

**Background:**

Tuberculosis (TB) remains a significant health concern, contributing to the highest mortality among infectious diseases worldwide. However, none of the various TB diagnostic tools introduced is deemed sufficient on its own for the diagnostic pathway, so various artificial intelligence (AI)–based methods have been developed to address this issue.

**Objective:**

We aimed to provide a comprehensive evaluation of AI-based algorithms for TB detection across various data modalities.

**Methods:**

Following PRISMA (Preferred Reporting Items for Systematic Reviews and Meta-Analysis) 2020 guidelines, we conducted a systematic review to synthesize current knowledge on this topic. Our search across 3 major databases (Scopus, PubMed, Association for Computing Machinery [ACM] Digital Library) yielded 1146 records, of which we included 152 (13.3%) studies in our analysis. QUADAS-2 (Quality Assessment of Diagnostic Accuracy Studies version 2) was performed for the risk-of-bias assessment of all included studies.

**Results:**

Radiographic biomarkers (n=129, 84.9%) and deep learning (DL; n=122, 80.3%) approaches were predominantly used, with convolutional neural networks (CNNs) using Visual Geometry Group (VGG)-16 (n=37, 24.3%), ResNet-50 (n=33, 21.7%), and DenseNet-121 (n=19, 12.5%) architectures being the most common DL approach. The majority of studies focused on model development (n=143, 94.1%) and used a single modality approach (n=141, 92.8%). AI methods demonstrated good performance in all studies: mean accuracy=91.93% (SD 8.10%, 95% CI 90.52%-93.33%; median 93.59%, IQR 88.33%-98.32%), mean area under the curve (AUC)=93.48% (SD 7.51%, 95% CI 91.90%-95.06%; median 95.28%, IQR 91%-99%), mean sensitivity=92.77% (SD 7.48%, 95% CI 91.38%-94.15%; median 94.05% IQR 89%-98.87%), and mean specificity=92.39% (SD 9.4%, 95% CI 90.30%-94.49%; median 95.38%, IQR 89.42%-99.19%). AI performance across different biomarker types showed mean accuracies of 92.45% (SD 7.83%), 89.03% (SD 8.49%), and 84.21% (SD 0%); mean AUCs of 94.47% (SD 7.32%), 88.45% (SD 8.33%), and 88.61% (SD 5.9%); mean sensitivities of 93.8% (SD 6.27%), 88.41% (SD 10.24%), and 93% (SD 0%); and mean specificities of 94.2% (SD 6.63%), 85.89% (SD 14.66%), and 95% (SD 0%) for radiographic, molecular/biochemical, and physiological types, respectively. AI performance across various reference standards showed mean accuracies of 91.44% (SD 7.3%), 93.16% (SD 6.44%), and 88.98% (SD 9.77%); mean AUCs of 90.95% (SD 7.58%), 94.89% (SD 5.18%), and 92.61% (SD 6.01%); mean sensitivities of 91.76% (SD 7.02%), 93.73% (SD 6.67%), and 91.34% (SD 7.71%); and mean specificities of 86.56% (SD 12.8%), 93.69% (SD 8.45%), and 92.7% (SD 6.54%) for bacteriological, human reader, and combined reference standards, respectively. The transfer learning (TL) approach showed increasing popularity (n=89, 58.6%). Notably, only 1 (0.7%) study conducted domain-shift analysis for TB detection.

**Conclusions:**

Findings from this review underscore the considerable promise of AI-based methods in the realm of TB detection. Future research endeavors should prioritize conducting domain-shift analyses to better simulate real-world scenarios in TB detection.

**Trial Registration:**

PROSPERO CRD42023453611; https://www.crd.york.ac.uk/PROSPERO/view/CRD42023453611

## Introduction

Tuberculosis (TB) stood as the dominant infectious disease threat worldwide, affecting over 10.5 million individuals and claiming the lives of 1.3 million people in 2022 [1]. A pivotal strategy in halting this worldwide epidemic revolves around breaking the transmission chain by identifying and treating all individuals with infectious forms of TB [2]. Various diagnostic tools have been introduced, including chest radiography, tuberculin skin tests, interferon-gamma release assays, sputum smear microscopy, sputum mycobacterial culture, and an array of nucleic acid amplification tests [3]. Nonetheless, each of these diagnostic modalities faces significant implementation challenges, and none alone is deemed adequate for the diagnostic pathway. Consequently, researchers have endeavored to integrate artificial intelligence (AI)–based algorithms to augment the detection of TB.

AI encompasses a spectrum of techniques enabling computer programs to tackle intricate problems by emulating human cognitive processes [4]. Presently, 2 widely used terms denote AI techniques that necessitate minimal-to-no human intervention: machine learning (ML) and deep learning (DL). ML possesses the capability to discern meaningful patterns within datasets autonomously, without explicit programming [5]. Conversely, DL emerges as a subdomain within ML, leveraging deep and intricate neural networks to extract features or patterns from datasets for subsequent analysis [5,6].

In this study, we aimed to comprehensively evaluate the performance of AI-based algorithms, particularly ML and DL, for TB detection. This aligns closely with the first pillar of the World Health Organization (WHO) End TB Strategy, which includes systematic screening for TB in high-risk groups [7]. In fact, a novel recommendation was issued by WHO in 2021: the approval of AI tools to analyze chest X-rays (CXRs) for TB detection in place of human readers [8].

Several notable review papers have emerged. Jimmy et al [9] conducted a meticulous systematic review, delving into various DL methods for TB detection based on CXR. Similarly, Sharma et al [10] contributed a narrative survey spotlighting DL-based convolutional neural networks (CNNs) tailored for TB diagnosis from CXR. Zeyu et al [11] embarked on a narrative review aimed at dissecting diverse DL-based approaches for TB diagnosis. Oloko-Oba and Viriri [12] scrutinized DL techniques for TB detection via chest radiographs. Santosh et al [13] conducted a systematic review, albeit with a narrower time frame spanning 5 years (2016-2021), focusing on DL for TB screening using CXR. Carvalho et al [14] contributed a systematic review on DL techniques used for classifying TB bacilli in microscopic Ziehl-Neelsen (ZN) sputum smear images. Da Silva Barros et al [15] conducted a systematic review on ML models geared toward predicting TB treatment outcomes. Distinguished from these endeavors, our current review took a broader perspective, encompassing not only DL but also ML as integral components of AI-based methods for TB detection.

Siddiqui and Garg [16] examined recent studies of intelligent techniques (ML and DL) for diagnosing pulmonary TB. Singh et al [17] scrutinized the drawbacks of conventional TB diagnostics, while exploring the utility of ML and DL methods for TB diagnosis. Additionally, they highlighted several commercial computer-aided detection (CAD) tools as promising AI-driven instruments. Notably, both reviews were reported as narrative reviews, rather than using the systematic review methodology we adopted here.

Harris et al [18] meticulously conducted a systematic review focusing on the diagnostic accuracy of AI-based tools in TB detection using CXR. Hansun et al [19] recently published a systematic review examining the effectiveness of ML and DL methods for TB detection using CXR. Both studies aligned closely with our review but were limited to CXR. In contrast, our review encompassed a broader spectrum of diagnostic data, including radiographic, biochemical, physiological, and other clinical data. This broader scope enabled a more comprehensive evaluation of AI-based methods across diverse data modalities for TB detection.

## Methods

### Design, Registration, and Information Sources

In conducting this systematic review, we adhered to the PRISMA (Preferred Reporting Items for Systematic Reviews and Meta-Analyses) 2020 guidelines (Multimedia Appendix 1) [20]. Our review protocol, in accordance with the PRISMA-Protocol 2015 statement [21,22], was registered on PROSPERO (Prospective Register of Systematic Reviews; ID CRD42023453611) [23,24].

The systematic review drew upon 3 primary academic search systems and online databases: Scopus, PubMed, and the Association for Computing Machinery (ACM) Digital Library. These platforms are widely recognized as pivotal sources for comprehensive literature reviews [25]. The searches encompassed all published literature up to July 25, 2023.

### Ethical Considerations

As this systematic literature review focused on retrospective studies, no ethical approval was required.

### Search Strategy

For our search strategy, we used 3 primary keywords: “artificial intelligence,” “tuberculosis,” and “detect*.” Additionally, we incorporated several alternative terms for each main keyword to ensure comprehensive coverage during the search process, resulting in the findings outlined in Table 1. Initially, we identified a total of 1146 records; however, certain records were not available from the respective databases (n=58, 5.1%). The full search queries can be seen in Multimedia Appendix 2.

**Table 1:** Sample search queries and results.

Search query	Scopus, n/N (%)	PubMed, n/N (%)	ACM^a^, n/N (%)
(TITLE-ABS-KEY (“Machine Learning” OR “Predictive Analytics” OR “Statistical Learning” OR “Deep Learning” OR “Artificial Intelligence” OR “AI”) AND TITLE-ABS-KEY (“Tuberculosis” OR “TB”) AND TITLE-ABS-KEY (“Early detection” OR “detect*” OR “Early diagnosis” OR “diagnosis”)) AND DOCTYPE (ar OR cp) AND ( LIMIT-TO (LANGUAGE , “English”)) AND (LIMIT-TO (SRCTYPE , “j”) OR LIMIT-TO (SRCTYPE , “p”))	857/1146 (74.8)	284/1146 (24.8)	5/1146 (0.4)
Not available for download (n=58)	53/58 (91.4)	5/58 (8.6)	0
Available for download (n=1088)	804/1088 (73.9)	279/1088 (25.6)	5/1088 (0.5)

^a^ACM: Association for Computing Machinery.

### Inclusion and Exclusion Criteria, Data Extraction, and Storage

All downloaded records underwent scrutiny against the inclusion and exclusion criteria outlined for this review study. There were 4 criteria that all included studies had to meet: (1) full-text papers reporting original data published in peer-reviewed journals or proceedings, (2) cross-sectional diagnostic test evaluations, (3) papers focused on TB detection using AI, and (4) papers written in English.

The information extracted from each included study comprised the title, authors, publication year, journal title, objectives, and outcome measures. Dataset characteristics, including their source and total number of data items, were documented, along with details regarding the modality and type of data used. The AI methods applied, evaluation techniques used, performance metrics, best results obtained, and any comparisons with other studies were recorded. Additionally, outcome types, citation counts, and information regarding sponsors or funding sources, were also gathered as part of the comprehensive extraction process.

Each review author was actively engaged in the conceptualization and execution of all phases outlined in the PRISMA 2020 guidelines. At least 2 review authors were involved in each phase, including identification, screening, and assessment of eligibility for inclusion. To ensure accuracy and transparency, we documented the results of each phase using a standardized spreadsheet.

### Outcomes Assessed

The first outcome from this review was the compilation of diverse AI methods used for TB detection. The second outcome encompassed diagnostic performance exhibited by various AI methods in TB detection. This summary encompassed key metrics, such as accuracy, area under the receiver operating characteristic (ROC) curve (AUROC), sensitivity, and specificity, providing valuable insights into the efficacy and reliability of these methods in clinical practice.

### Strategy for Data Analysis and Synthesis

We adopted a narrative synthesis approach, integrating information extracted from the included studies within the text. The narrative synthesis method facilitated an in-depth exploration of the relationships and insights both within and across included studies, stratified by various attributes, such as modality, biomarker types, and AI methods. Descriptive statistics, primarily using box-whisker and violin plots, alongside tables and figures, presented the quantitative outcomes of this systematic review. Given the pronounced heterogeneity observed in study designs, comparators, biomarker types, evaluation techniques, and AI methods encompassed in this review study, a meta-analysis was deemed impractical.

### Risk-of-Bias Assessment

In assessing the risk of bias within the included studies, we used the QUADAS-2 (Quality Assessment of Diagnostic Accuracy Studies version 2) tool. This tool is widely recommended for systematic reviews as it enables the evaluation of bias and applicability in diagnostic accuracy studies [26]. It comprises 4 key domains: (1) patient selection, (2) index test, (3) reference standard, and (4) flow and timing. To tailor the assessment to the specific focus of this review on evaluating AI-based methods’ performance in TB detection, adjustments were made to the questions in the QUADAS-2 tool. Two review authors (SH and AW) assessed each included study, and a third opinion was sought from the author AA or the author GBM in the case of disagreements.

## Results

### Overview

**Figure 1:**
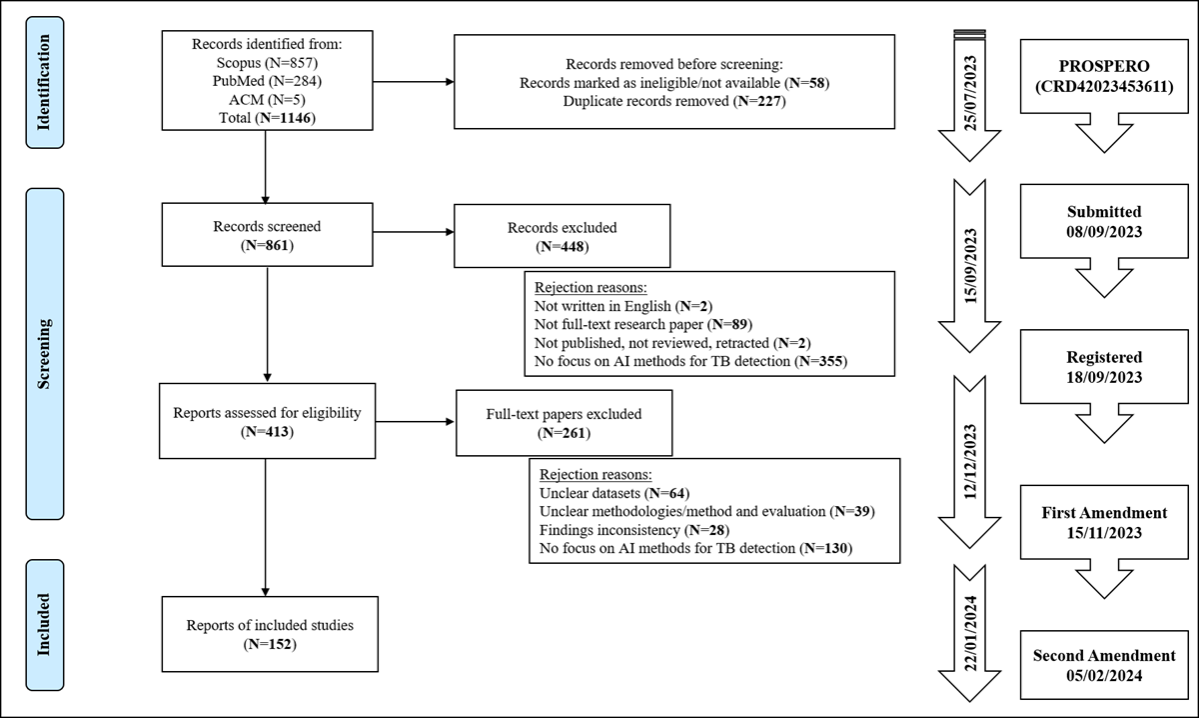
PRISMA 2020 flowchart with timeline and PROSPERO information. ACM: Association for Computing Machinery; AI: artificial intelligence; PRISMA: Preferred Reporting Items for Systematic Reviews and Meta-Analysis; TB: tuberculosis.

Figure 1 delineates all the processes conducted throughout the systematic review. We adhered to the PRISMA 2020 flowchart, including the incorporation of a process timeline and the inclusion of a registered protocol status in PROSPERO.

Of the 1146 records initially identified from Scopus (n=857, 74.8%), PubMed (n=284, 24.8%), and ACM Digital Library (n=5, 0.4%), 58 (5.1%) were found to be unavailable. Upon checking and removing duplicates (n=227, 20.9%), we were left with 861 (79.1%) unique titles to be passed to the screening phase. Subsequently, titles, abstracts, and keywords underwent examination based on 4 primary selection criteria, where 413 (48%) records remained for eligibility assessment.

All 413 records underwent thorough assessment by examining their full-text content. From this pool, 261 (63.2%) papers were excluded, leaving us with a total of 152 (36.8%) papers being included as part of this systematic review. Subsequently, the data extraction process was carried out for all included studies. The entirety of the review process spanned approximately 6 months (July 2023-January 2024).

### General Characteristics of Included Studies

A summary of the general characteristics of the 152 studies included in this systematic review is included in Tables 2 and 3. The majority of the included studies opted for a single modality (n=141, 92.8%), with a smaller subset using a multimodal approach (n=11, 7.2%). Among the various data types used, radiographic data emerged as the most prevalent data type (n=129, 84.9%), followed by biochemical (n=21, 13.8%) and physiological (n=16, 10.5%) data types. Several studies using a multimodal approach integrated more than 1 data type, such as a combination of radiographic and physiological data types.

**Table 2:** Summary of mutually exclusive general characteristics of the included studies (N=152).

Characteristics		Studies, n (%)
**Modality**		
	Single	141 (92.8)
	Multimodal	11 (7.2)
**Evaluation technique**		
	Holdout	95 (62.5)
	k-Fold CV^a^	48 (31.6)
	External data	9 (5.9)
**Outcome type**		
	Model	143 (94.1)
	Application/prototype	8 (5.3)
	Clinical evaluation	1 (0.7)
TL^b^		89 (58.6)
Comparison with other studies		68 (44.7)
Funding/sponsor		78 (51.3)

^a^CV: cross-validation.

^b^TL: transfer learning.

**Table 3:** Summary of nonmutually exclusive general characteristics of the included studies (N=152).

Characteristics		Studies, n (%)
**AI^a^ type**		
	ML^b^	49 (32.2)
	DL^c^	122 (80.3)
**Reference standard**		
	Human reader	103 (67.8)
	Bacteriological	43 (28.3)
	Not available	24 (15.8)
**Type of data**		
	Radiographic	129 (84.9)
	Biochemical	21 (13.8)
	Physiological and clinical	16 (10.5)

^a^AI: artificial intelligence.

^b^ML: machine learning.

^c^DL: deep learning.

In terms of evaluation techniques, the majority of studies (n=95, 62.5%) used the holdout evaluation technique, which involves dividing the entire dataset into training and test sets. In total, 48 (31.6%) studies used the k-fold cross-validation (CV) technique, dividing the dataset into k partitions and conducting the evaluation k times. Only 9 (5.9%) studies incorporated external datasets during the evaluation phase. Of the 152 included studies, 143 (94.1%) focused on AI model development, while 8 (5.3%) studies concentrated on application or prototype creation, and 1 (0.7%) study examined the clinical evaluation of AI-based CAD tools.

Regarding the AI approaches, a large number of studies (n=122, 80.3%) used various DL methods, while 49 (32.2%) studies used ML methods. We also collected information regarding various reference standards used in the included studies. We classified them into 2 groups, namely the human reader and bacteriological standards. Of the 152 included studies, 103 (67.8%) used human reader reference standards, 43 (28.3%) used bacteriological reference standards, and 24 (15.8%) did not report any reference standard. Similar to the data-types group, both AI-type and reference standard groups were not mutually exclusive.

In addition, 89 (58.6%) studies used the TL approach, a relatively recent and validated strategy for addressing domain-specific challenges [27], especially prevalent in studies using DL methods. Moreover, 68 (44.7%) studies conducted comparisons with multiple prior studies. Notably, an equal proportion of studies received funding or sponsorship from various entities (n=78, 51.3%) compared to those that did not receive any funding (n=74, 48.7%). All included studies were published between 2011 and 2023.

A total of 141 distinct data sources were identified across the included studies, comprising 2,072,457 records. Among these, the most frequently used data sources were Shenzhen (SZ; n=51, 36.2%), Montgomery County (MC; n=46, 32.6%), Kaggle TB CXR (n=6, 4.3%), and TBX11K (n=5, 3.5%), all of which provided radiographic data. MC and SZ were the predominant data sources, both publicly available CXR datasets from the US National Library of Medicine. MC contains 138 images, while SZ offers 662 images [28]. The Kaggle TB CXR dataset contains 7000 images, although only 4200 (60%) are publicly accessible [29]. Conversely, TBX11K, introduced in 2020, stands out as a newer and more extensive CXR dataset for TB-related research, featuring a total of 11,200 images [30]. For further insights into the extracted data from all included studies, detailed information is available in Multimedia Appendix 3.

### AI Techniques for TB Detection

Among the 152 included studies, the majority were published within the past 8 years, with only 3 (2%) studies predating 2016 [31-33]. As of January 18, 2024, these studies collectively garnered 6070 citations on Google Scholar. Remarkably, the top 3 (2%) cited publications were identified as Lakhani and Sundaram [34] with 1695 citations, Pasa et al [35] with 312 citations, and Lopes and Valiati [36] with 260 citations. This underscored the burgeoning interest among researchers in integrating AI-based methods into TB detection studies.

The included studies implemented various AI-based algorithms, which could be broadly categorized into 2 major approaches: ML and DL. Specifically, 102 (67.1%) studies used DL methods [30,34,35,37-135], while 30 (19.7%) studies used ML techniques [31-33,136-162]. Additionally, 19 (12.5%) studies used a combination of both ML and DL approaches [36,163-180]. Furthermore, 1 (0.7%) study [181] focused on the implementation of a DL-based CAD tool, namely Lunit INSIGHT v4.7.2, specifically designed for systematic TB screening in a low-prevalence setting.

**Figure 2:**
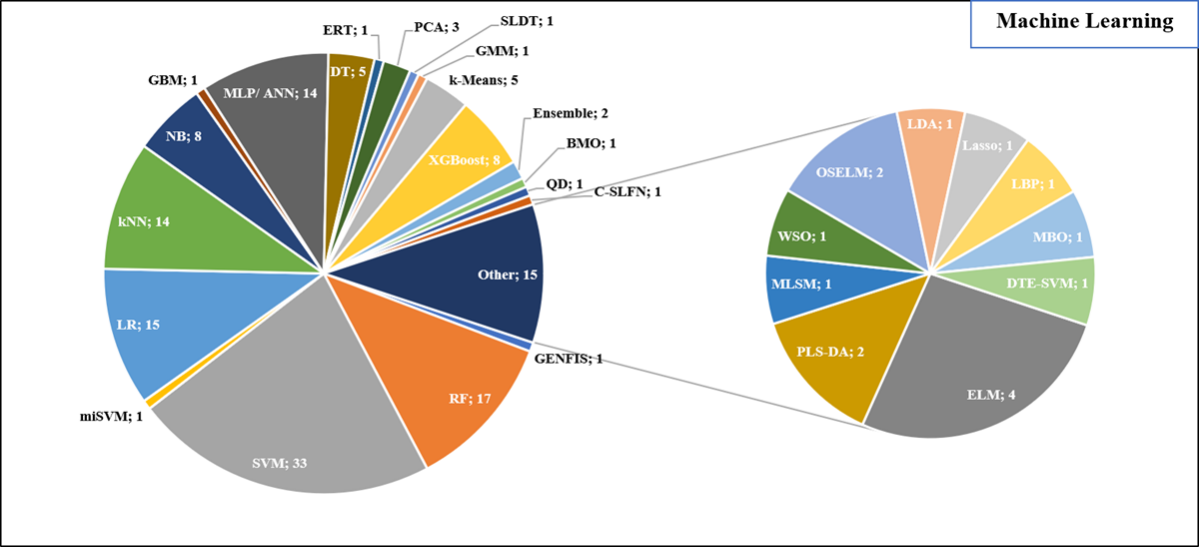
Different ML methods identified from the included studies. ANN: artificial neural network; BMO: bird mating optimizer; DT: decision tree; DTE-SVM: deep transferred EfficientNet with support vector machine; ELM: extreme learning machine; ERT: extremely randomized tree; GBM: Generalized Boosting Machine; GENFIS: genetic-neuro-fuzzy inferential system; GMM: Gaussian mixture model; kNN: k nearest neighbor; Lasso: least absolute shrinkage and selection operator; LBP: local binary pattern; LDA: linear discriminant analysis; LR: logistic regression; MBO: monarch butterfly optimization; ML: machine learning; MLP: multilayer perceptron; MLSM: multilevel similarity measure; NB: naïve Bayes; OSELM: online sequential extreme learning machine. PLS-DA: partial least squares–discriminant analysis; PCA: principal component analysis; QD: quadratic discriminant; RF: random forest; SLDT: stacked loopy decision tree; SLFN: single hidden layer feedforward neural network; SVM: support vector machine; WSO: water strider optimization; XGBoost: extreme gradient boosting.

Figure 2 illustrates the diverse array of ML methods used in the included studies. Notably, support vector machines (SVMs; n=33, 21.7%), random forests (RFs; n=17, 11.2%), and logistic regression (LR; n=15, 9.9%) emerged as the 3 most prevalent ML techniques.

**Figure 3:**
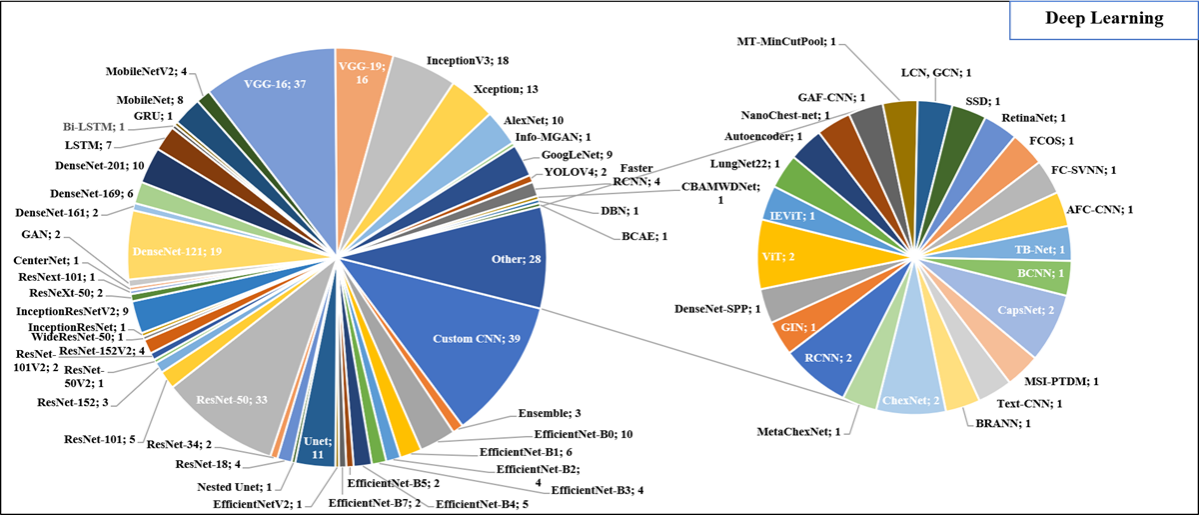
DL methods identified from the included studies. AFC-CNN: adaptive fractional crow convolutional neural network; BCNN: Bayesian-based convolutional neural network; bi-LSTM: bidirectional long short-term memory; BRANN: Bayesian regularization artificial neural network; CBAM: convolutional block attention module; CNN: convolutional neural network; DenseNet-SPP: DenseNet with spatial pyramid pooling; DL: deep learning; FC-SVNN: fractional crow search–based deep convolutional neural network; FCOS: fully convolutional one stage; GAF-CNN: Gramian angular field convolutional neural network; GAN: generative adversarial network; GCN: global complex network; GIN: graph isomorphism network; GRU: gated recurrent unit; IEViT: image enhanced vision transformer; LCN: local complex network; LSTM: long short-term memory; MSI-PTDM: multistream integration–pulmonary tuberculosis diagnosis model; MT-MinCutPool: multivariate time series with MinCutPool; RCNN: region-based convolutional neural network; SSD: single-shot multibox detector; VGG: Visual Geometry Group; ViT: vision transformer; YOLO: you only look once.

CNNs were the predominant DL method among the included studies. Alongside custom CNNs (n=39, 25.7%), many researchers have leveraged established DL architectures, notably including Visual Geometry Group (VGG)-16 (n=37, 24.3%), ResNet-50 (n=33, 21.7%), and DenseNet-121 (n=19, 12.5%). Figure 3 provides an overview of the various DL methods identified in the included studies.

Regarding the diagnostic performance of AI methods used in the literature, the most frequently used evaluation metrics encompassed accuracy (n=128, 84.2%), sensitivity (n=112, 73.7%), the AUC (n=87, 57.2%), and specificity (n=77, 50.7%). Accuracy denotes the proportion of correctly predicted cases, encompassing true positives and true negatives among all cases [182]. Sensitivity measures a model’s ability to accurately identify individuals with a condition [183,184]. The AUC, derived from the ROC curve, quantitatively assesses a model’s performance [182]. Specificity gauges a model’s accuracy in identifying individuals without a condition [183,184]. Table 4 presents the distribution of various performance metrics identified in the included studies. For an in-depth exploration of the performance metrics used across the included studies, refer to the detailed description provided in Multimedia Appendix 4.

**Table 4:** Different performance evaluation metrics in the included studies (N=152).

Metric	Studies, n (%)
Accuracy	128 (84.2)
AUROC^a^	87 (57.2)
Sensitivity/recall/true-positive rate	112 (73.7)
Specificity/true-negative rate	77 (50.7)
Precision/positive predictive value	56 (36.8)
Negative predictive value	9 (5.9)
*F*_1_-score	55 (36.2)
Average recall	1 (0.7)
Mean average precision	2 (1.3)
Matthews correlation coefficient	7 (4.6)
True detection rate	1 (0.7)
Area under the alternative free-response ROC^b^ curve	1 (0.7)
Error loss	1 (0.7)
Cohen kappa	3 (2.0)
Root mean square error	2 (1.3)
False positive rate	4 (2.6)
False negative rate	1 (0.7)
Squared error	1 (0.7)
Balanced accuracy	1 (0.7)
Fowlkes-Mallows index	1 (0.7)

^a^AUROC: area under the receiver operating characteristic curve.

^b^ROC: receiver operating characteristic.

### AI Diagnostic Performance

**Figure 4:**
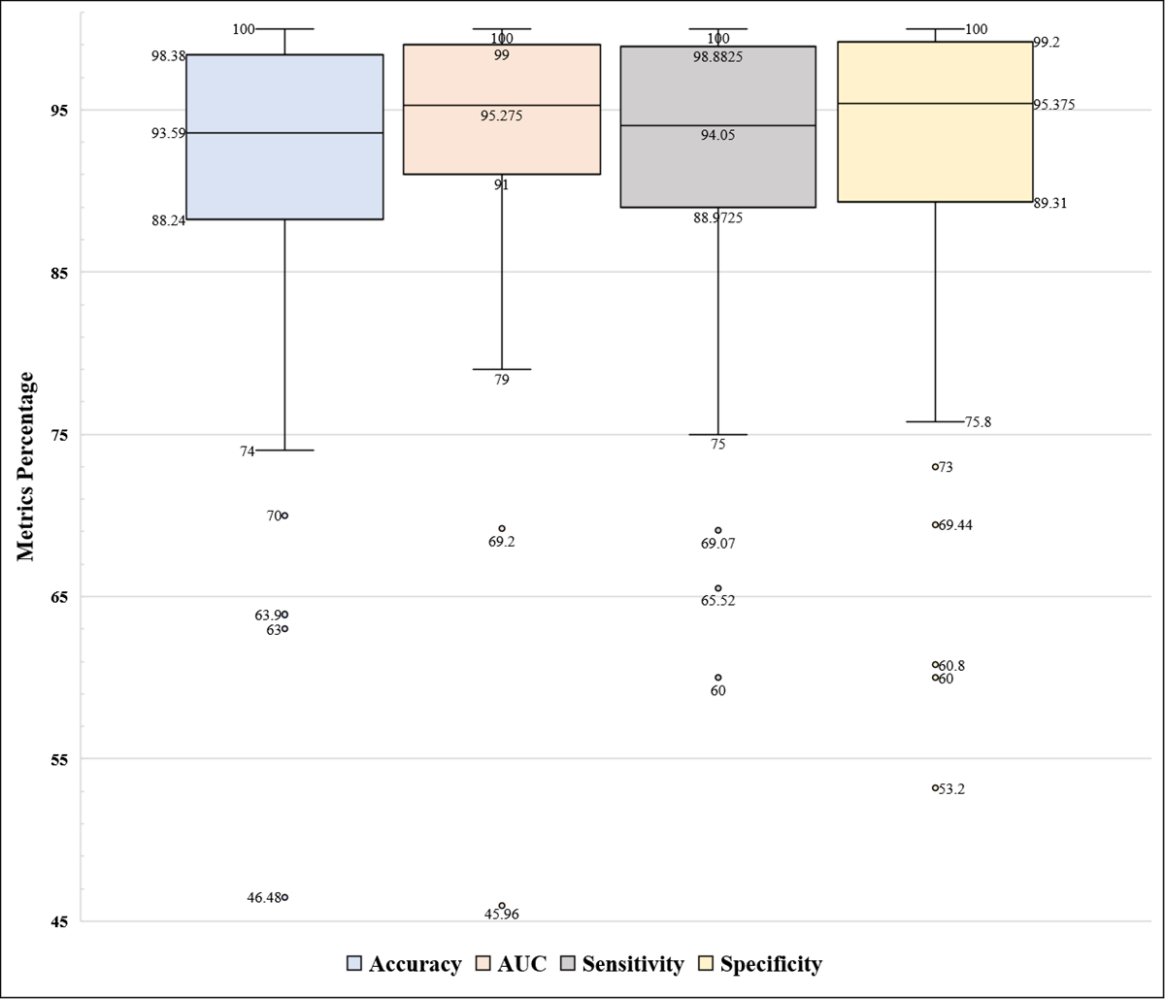
Overall accuracy, AUC, sensitivity, and specificity of the included studies. AUC: area under the curve.

Figure 4 provides a comprehensive overview of the overall performance results across all included studies. From the depicted box plot, it is evident that accuracy ranged from 46.48% [59] to 100% [118], with a mean value of 91.93% (SD 8.10%, 95% CI 90.52%-93.33%) and a median of 93.59% (IQR 88.33%-98.32%). Similarly, the AUC ranged from 45.96% [59] to 100% [72,78,96,178], with a mean value of 93.48% (SD 7.51%, 95% CI 91.90%-95.06%) and a median of 95.28% (IQR 91%-99%). Sensitivity spanned from 60% [156] to 100% [85,92,100,118,128,132,141,146,163,178,181], with a mean value of 92.77% (SD 7.48%, 95% CI 91.38%-94.15%) and a median of 94.05% (IQR 89%-98.87%). Meanwhile, specificity ranged from 53.2% [129] to 100% [113,162,174,177], with a mean value of 92.39% (SD 9.4%, 95% CI 90.30%-94.49%) and a median of 95.38% (IQR 89.42%-99.19%). Several outliers were detected for each metric, including 4 for accuracy, at 46.48% [59], 63% [126], 63.9% [129], and 70% [156]; 2 for the AUC, at 45.96% [59] and 69.2% [129]; 3 for sensitivity, at 60% [156], 65.52% [155], and 69.07% [143]; and 5 for specificity, at 53.2% [129], 60% [146], 60.8% [168], 69.44% [67], and 73% [152].

**Figure 5:**
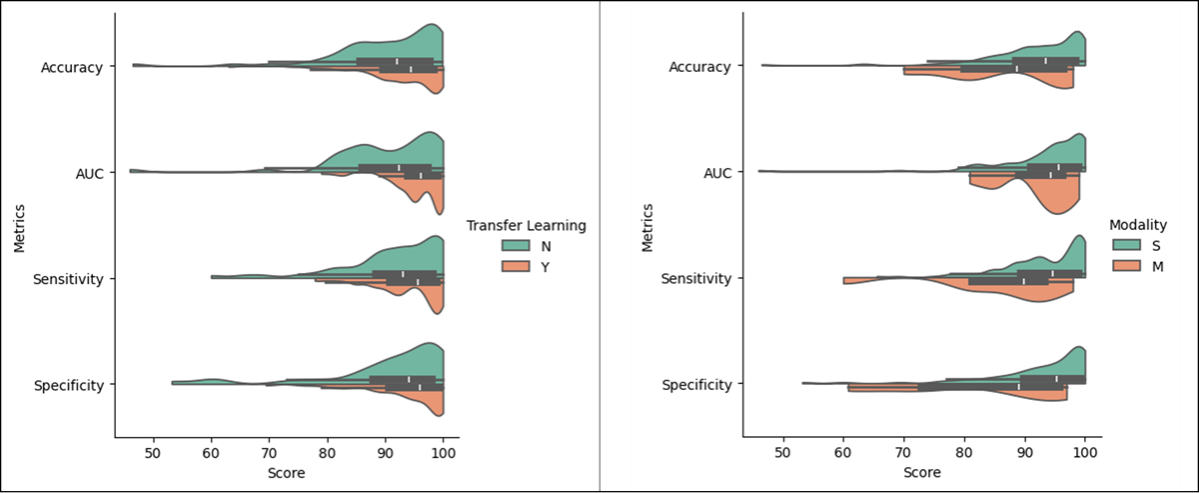
Comparative performance based on TL (left) and modality (right). AUC: area under the curve; M: multimodal; N: no; S: single; TL: transfer learning; Y: yes.

We conducted further performance comparisons based on several criteria, including whether a study used the TL method and whether it adopted a single (S) or multimodal (M) approach. Figure 5 presents split-grouped violin plots illustrating the comparative performance results of the included studies based on TL (yes [Y], no [N]) and the modality used.

**Figure 6:**
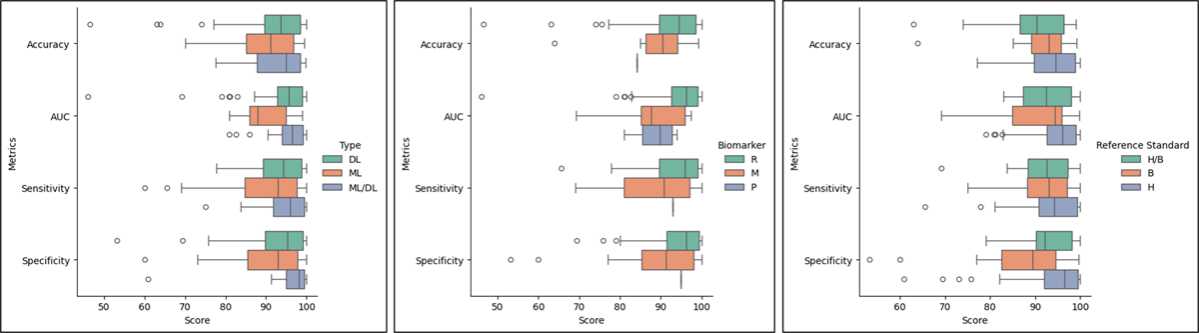
Comparative performance based on AI methods (left), biomarker types (middle), and reference standards (right). AI: artificial intelligence; AUC: area under the curve; B: bacteriological; DL: deep learning; H: human; H/B: both human and bacteriological; M: molecular; ML: machine learning; ML/DL: both machine learning and deep learning; P: physiological; R: radiographic.

We also examined the performance of AI methods used in all the included studies based on the AI techniques applied (ML, DL, or both [ML/DL]), the types of data or biomarkers used (radiographic [R], molecular [M], physiological [P]), and the reference standard used (human [H], bacteriological [B], or both [H/B]). Figure 6 showcases the box plots, offering a comparative analysis of the performance results of the included studies based on AI methods, biomarker types, and reference standards.

Specifically, the AI performance across different biomarker types showed mean accuracies of 92.45% (SD 7.83%), 89.03% (SD 8.49%), and 84.21% (SD 0%); mean AUCs of 94.47% (SD 7.32%), 88.45% (SD 8.33%), and 88.61% (SD 5.9%); mean sensitivities of 93.8% (SD 6.27%), 88.41% (SD 10.24%), and 93% (SD 0%); and mean specificities of 94.2% (SD 6.63%), 85.89% (SD 14.66%), and 95% (SD 0%) for radiographic, molecular/biochemical, and physiological biomarkers, respectively. Meanwhile, AI performance across various reference standards show mean accuracies of (SD 7.3%), 93.16% (SD 6.44%), 88.98% (SD 9.77%); mean AUCs of 90.95% (SD 7.58%), 94.89% (SD 5.18%), 92.61% (SD 6.01%); mean sensitivities of 91.76% (SD 7.02%), 93.73% (SD 6.67%), 91.34% (SD 7.71%); and mean specificities of 86.56% (SD 12.8%), 93.69% (SD 8.45%), 92.7% (SD 6.54%) for bacteriological, human reader, and combined bacteriological and human reader reference standards, respectively.

### Risk-of-Bias Assessment Result

**Figure 7:**
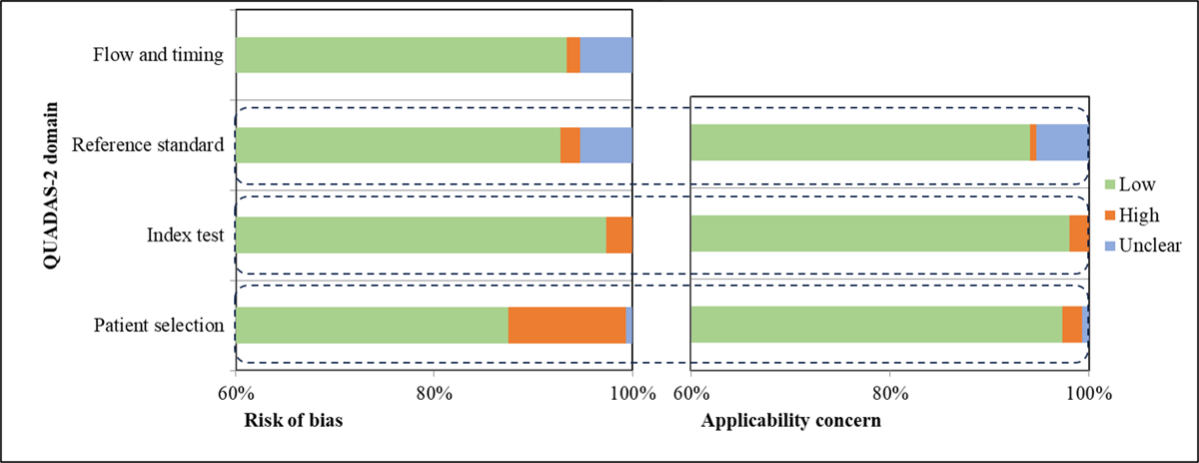
Summary of QUADAS-2 results of the included studies. QUADAS-2: Quality Assessment of Diagnostic Accuracy Studies version 2.

Figure 7 presents the QUADAS-2 outcomes across all included studies. Among the assessed studies, 18 (11.8%) [32,46-48,57,70,95,97,111,124,127,136,140,144,153,169,170,178] were flagged for a high risk of bias, with 1 (0.7%) study [141] marked as having an unclear risk concerning patient selection, largely attributable to incomplete or absent information in the data selection process. Moreover, 4 (2.6%) studies [41,101,108,174] were identified as having a high risk regarding the index test, attributed to the absence of crucial details on model architecture and parameters. Additionally, 3 (2%) studies [57,156,158] were classified as having a high risk and 8 (5.3%) studies [32,63,64,97,98,111,140,161] as having an unclear risk concerning reference standards, due to incomplete or missing information regarding the reference standards used. Concerning flow and timing, 2 (1.3%) studies [88,95] were deemed to have a high risk of bias, while 8 (5.3%) studies [53,65,67,87,102,141,156,179] were assigned an unclear risk, primarily due to unclear or absent information regarding the time interval and intervention provided in those studies.

The majority of the included studies exhibited a low level of concern about applicability. Only 3 (2%) studies [48,88,144] were identified with a high risk and 1 (0.7%) study [141] with an unclear risk in terms of patient selection. Additionally, 3 (2%) studies [41,108,151] were flagged with a high risk concerning the index test, while 1 (0.7%) study [158] showed a high risk, and 8 (5.3%) studies [32,63,64,97,98,111,140,161] had an unclear risk concerning reference standards. For comprehensive QUADAS-2 results, please refer to Multimedia Appendix 5.

## Discussion

### Principal Findings

This review study examined the diagnostic performance of various AI-based algorithms, including both ML and DL, for TB detection across different techniques, data modalities, and reference standards used in the included studies. Approximately 84.9% (n=129) of the included studies used radiographic data types, particularly CXRs. Notably, the SZ and MC datasets emerged as the most frequently used, with 51 and 46 studies, respectively, making use of them. However, it is noteworthy that the availability of data in SZ and MC, totaling 800 images, is relatively limited, posing challenges, especially for studies using DL methods where larger datasets are often required for optimal performance. Consequently, numerous techniques have been introduced to augment data quantity [185,186], including conventional methods, as well as DL-based augmentation techniques.

Few recent studies have used other data types, such as exhaled breath particles [146,150] and cough sounds [63,64,175]. Although not recommended as a standard tool for TB detection, results from these noninvasive approaches could be a supplement to the standard TB detection tool using CXRs. Hence, further studies that analyze CXRs together with other data types are encouraged.

Concerning modality, the majority of the included studies adopted a single modality (n=141, 92.8%) rather than using a multimodal approach (n=11, 7.2%). Studies using a single modality tended to exhibit higher median values and narrower distribution ranges compared to those using a multimodal approach (Figure 5, right). This suggests that the single-modality approach may outperform the multimodal approach. However, more outliers were observed in all performance metrics for the single modality compared to the multimodal approach. Additionally, these outcomes were not obtained under identical scenario settings. When applied under similar scenario settings, the multimodal approach has demonstrated the potential to enhance performance results compared to the single modality [9,187]. This is supported by findings from several included studies that have directly compared single-modality and multimodal approaches [79,86,95].

DL emerged as a cornerstone in the majority of the included studies (n=122, 80.3%), with CNNs taking the forefront. Among these, architectures such as VGG-16 [188], ResNet-50 [189,190], and DenseNet-121 [191] have gained notable traction. More recent DL architectures, including EfficientNets [192], Vision Transformer (ViT) [193], and ConvNeXts [194], have gained increasing attention. These emerging models, with more efficient architectures that require fewer computational resources, show potential for application in medical image processing, particularly in TB detection tasks.

Some of the included studies explored various explainable AI (XAI) methods to bolster the interpretability and trustworthiness of their AI models’ outcomes. Originating from the need to strike a balance between interpretability and accuracy in ML and DL models [195], XAI has garnered considerable attention. Among the XAI techniques used in the included studies were gradient-weighted class activation mapping (Grad-CAM), local interpretable model-agnostic explanation (LIME), and Shapley additive explanations (SHAP).

TL is a powerful technique that capitalizes on knowledge acquired from a source dataset and adapts it to a target domain, relaxing the assumption that training data must be independent and identically distributed with test data [196]. TL proves especially beneficial in scenarios with a limited data volume. Indeed, TL has been extensively used in the studies encompassed in this systematic review (n=89, 58.6%), particularly those using DL methods. Notably, as illustrated in Figure 5 (left), investigations integrating TL tend to exhibit a higher median accuracy compared to counterparts. Additionally, they showcase a narrower distribution range, suggesting that TL-based approaches consistently deliver superior and more consistent results.

The majority of the included studies (n=143, 94.1%) focused primarily on the development of AI models for TB detection. Only 8 studies [44,73,92,106,111,137,153,176] progressed beyond model development to build prototypes or systems aimed at supporting real-world applications. Among these, 3 studies implemented their proposed models in real-world settings: eRx, a mobile health system for TB diagnosis deployed in Lima, Peru [111]; the multistream integration–pulmonary tuberculosis diagnosis model (MSI-PTDM), multistream integration for a TB diagnosis model implemented in China [176]; and AIChest4All, an automated CXR-screening system used in Thailand [44]. We excluded studies evaluating commercial TB detection tools, such as Lunit INSIGHT, CAD4TB, and qXR, as they typically do not provide detailed discussions of the underlying AI models. For further insights into commercial TB detection tools, interested readers are referred to the narrative review by Singh et al [17].

In terms of overall performance metrics, the AI methods displayed remarkable achievements. Further analysis based on the AI methods used in the included studies revealed interesting insights. As illustrated in Figure 6 (left), DL exhibits higher accuracy with less variation compared to ML and ML/DL. However, regarding the AUC, sensitivity, and specificity, studies integrating ML/DL tend to yield superior results, followed by DL and then ML. This suggests that the fusion of ML and DL methods may enhance model performance, albeit few studies have explored this avenue.

We also examined the performance results concerning the types of data used. Radiographic data-type studies showcased superior median scores across all performance metrics (Figure 6, middle). Notably, they demonstrated a higher level of consistency, evidenced by their shorter IQRs in all performance metrics. Regarding the reference standards used in the included studies, studies that used the human reader exceled compared to those that used bacteriological and both reference standards, as indicated by the higher median and mean scores on all performance metrics in Figure 6 (right). Despite accuracy, the IQRs were also shorter for the AUC, sensitivity, and specificity. This finding might be rooted in the large number of included studies that used DL methods on radiographic data types with human reader reference standards.

Finally, it is worth noting that the majority of the included studies overlooked the evaluation of proposed solutions regarding the domain-shift problem. Remarkably, only 1 study [126] addressed domain-shift analysis in the context of TB detection, as highlighted by Hansun et al [197]. Although our review confirmed the high performance of AI-based methods across various data types in TB detection tasks, these evaluations were predominantly conducted on in-domain datasets. In other words, the datasets used for validation and testing shared the same distribution and characteristics as the training data. However, many AI methods, particularly DL, struggle when applied to real-world settings [198]. This discrepancy often arises due to differences in data distribution between the training data and real-world datasets. As such, it is imperative to broaden our focus to include not only in-domain evaluations but also domain-shift analysis in future research endeavors.

### Limitations and Strengths

This systematic review has several limitations that warrant acknowledgment. First, our search was limited to the literature published in English from 3 primary academic databases: Scopus, PubMed, and ACM Digital Library. Although these sources are comprehensive, it is possible that relevant studies might exist in other databases, potentially leading to a partial representation of the literature.

Second, we did not conduct further investigations into aspects with high or unclear risks from the bias assessment. Given the prevalence of radiographic datasets across most included studies, we assumed uniformity across these aspects. However, this assumption may overlook potential variations that could impact the overall assessment.

Lastly, in this review study, we opted not to perform a meta-analysis. Although a meta-analysis could provide valuable insights, the diverse nature of the included studies made it challenging to ensure meaningful comparability across studies, potentially affecting the validity and reliability of the synthesized findings. It is also important to note that the variations in data distribution and experimental settings across studies may lead to different results. Although the descriptive statistics reported in this study have been grouped based on several approaches, they should be seen as general performance results of AI for TB detection.

Despite the aforementioned limitations, our review study is among the few to systematically review the evidence available in the literature regarding the efficacy of AI-based methods for TB detection. Although most review studies focus only on a specific biomarker type for TB detection, we performed a more comprehensive review of AI-based methods across diverse biomarker types. We further assessed the AI methods’ performance based on several dominant approaches used in the included studies, including TL, multimodalities, reference standards, and ML/DL fusion methods for TB detection, which have never been explored before.

### Key Takeaways

AI-based approaches, particularly DL, have been extensively used for TB detection with high-accuracy results. A single-modality approach with chest radiographs has been dominantly used. Further studies that analyze chest radiographs together with other data types (multimodality) are encouraged. AI models trained and tested on radiographic data tend to achieve higher performance compared to other data types. Emerging DL models, such as EfficientNets, ViT, and ConvNeXts, show the potential to enhance TB detection results. TL has shown a great advantage in handling a limited data volume and consistently delivering superior results. Most of the included studies evaluated their proposed solutions using in-domain datasets. Future research endeavors should prioritize conducting domain-shift analyses to better simulate real-world scenarios in TB detection.

### Conclusion

This systematic review underscores the considerable promise of AI-based approaches in TB detection. Among the array of AI methods, DL emerges as the predominant choice. This preference for DL is attributed to its consistently robust performance, likely bolstered by the prevalence of studies using radiographic data. A notable observation across many studies is the use of relatively small datasets. Despite achieving commendable results, the potential of DL models could be further enhanced with larger and more diverse datasets.

Finally, although AI models demonstrate impressive performance on in-domain datasets, there is a notable gap in evaluating their robustness to domain shifts. Future research endeavors should prioritize conducting domain-shift analyses to better simulate real-world scenarios in TB detection. This approach would provide invaluable insights into the generalizability and applicability of AI-based methods beyond controlled settings.

## Appendix

Multimedia Appendix 1 PRISMA (Preferred Reporting Items for Systematic Reviews and Meta-Analysis) checklist.

Multimedia Appendix 2 Full search queries.

Multimedia Appendix 3 Full characteristics of the included studies.

Multimedia Appendix 4 Complete data summary.

Multimedia Appendix 5 QUADAS-2 (Quality Assessment of Diagnostic Accuracy Studies version 2) summary.

Multimedia Appendix 6 ChatGPT (Chat Generative Pretrained Transformer) transcript.

## Abbreviations

ACM: Association for Computing Machinery
AI: artificial intelligence
AUC: area under the curve
AUROC: area under the receiver operating characteristic curve
CAD: computer-aided detection
ChatGPT: Chat Generative Pretrained Transformer
CNN: convolutional neural network
CV: cross-validation
CXR: chest X-ray
DL: deep learning
MC: Montgomery County
ML: machine learning
PRISMA: Preferred Reporting Items for Systematic Reviews and Meta-Analysis
PROSPERO: Prospective Register of Systematic Reviews
QUADAS-2: Quality Assessment of Diagnostic Accuracy Studies version 2
ROC: receiver operating characteristic
SZ: Shenzhen
TB: tuberculosis
TL: transfer learning
VGG: Visual Geometry Group
ViT: Vision Transformer
WHO: World Health Organization
XAI: explainable AI

## Data Availability

All data generated or analyzed during this study are included in the published paper and its Supplementary and Figure/Appendix Files.
