# Supplementary material for: Diagnostic Performance of Artificial Intelligence–Based Methods for Tuberculosis Detection: Systematic Review
Source: J Med Internet Res. 2025 Mar 7;27:e69068. doi: 10.2196/69068 (PMC11928776; doi:10.2196/69068)
Supplement: Multimedia Appendix 2 [file jmir_v27i1e69068_app2.pdf]

Full Searched Queries

Scopus

(TITLE-ABS-KEY ("Machine Learning" OR "Predictive Analytics" OR "Statistical Learning" OR "Deep Learning" OR "Artificial Intelligence" OR "AI") AND TITLE-ABS-KEY ("Tuberculosis" OR "TB") AND TITLE-ABS-KEY ("Early detection" OR "detect*" OR "Early diagnosis" OR "diagnosis")) AND DOCTYPE (ar OR cp) AND (LIMIT-TO (LANGUAGE , "English")) AND (LIMIT-TO (SRCTYPE , "j") OR LIMIT-TO (SRCTYPE , "p"))

ACM Digital Library

[[Title: "machine learning"] OR [Title: "predictive analytics"] OR [Title: "statistical learning"] OR [Title: "deep learning"] OR [Title: "artificial intelligence"] OR [Title: "ai"]] AND [[Title: "tuberculosis"] OR [Title: "tb"]] AND [[Title: "early detection"] OR [Title: "detect*"] OR [Title: "early diagnosis"] OR [Title: "diagnosis"]]

[[Publication Title: "machine learning"] OR [Publication Title: "predictive analytics"] OR [Publication Title: "statistical learning"] OR [Publication Title: "deep learning"] OR [Publication Title: "artificial intelligence"] OR [Publication Title: "ai"]] AND [[Publication Title: "tuberculosis"] OR [Publication Title: "tb"]] AND [[Publication Title: "early detection"] OR [Publication Title: "detect*"] OR [Publication Title: "early diagnosis"] OR [Publication Title: "diagnosis"]]

[[Abstract: "machine learning"] OR [Abstract: "predictive analytics"] OR [Abstract: "statistical learning"] OR [Abstract: "deep learning"] OR [Abstract: "artificial intelligence"] OR [Abstract: "ai"]] AND [[Abstract: "tuberculosis"] OR [Abstract: "tb"]] AND [[Abstract: "early detection"] OR [Abstract: "detect*"] OR [Abstract: "early diagnosis"] OR [Abstract: "diagnosis"]]

[[Keywords: "machine learning"] OR [Keywords: "predictive analytics"] OR [Keywords: "statistical learning"] OR [Keywords: "deep learning"] OR [Keywords: "artificial intelligence"] OR [Keywords: "ai"]] AND [[Keywords: "tuberculosis"] OR [Keywords: "tb"]] AND [[Keywords: "early detection"] OR [Keywords: "detect*"] OR [Keywords: "early diagnosis"] OR [Keywords: "diagnosis"]]

PubMed

(("Machine Learning"[Title/Abstract] OR "Predictive Analytics"[Title/Abstract] OR "Statistical Learning"[Title/Abstract] OR "Deep Learning"[Title/Abstract] OR "Artificial Intelligence"[Title/Abstract] OR "AI"[Title/Abstract]) AND ("Tuberculosis"[Title/Abstract] OR "TB"[Title/Abstract]) AND ("Early detection"[Title/Abstract] OR "detect*"[Title/Abstract] OR "Early diagnosis"[Title/Abstract] OR "diagnosis"[Title/Abstract])) AND ((ffrft[Filter]) AND (fft[Filter]) AND (english[Filter]))
