# Supplementary material for: Diagnostic Performance of Artificial Intelligence–Based Methods for Tuberculosis Detection: Systematic Review
Source: J Med Internet Res. 2025 Mar 7;27:e69068. doi: 10.2196/69068 (PMC11928776; doi:10.2196/69068)
Supplement: Multimedia Appendix 6 [file jmir_v27i1e69068_app6.pdf]

user

improve this:

Tuberculosis (TB) was the leading infectious disease killer in the world prior to the COVID-19 pandemic with over 10.5 million people affected and 1.3 million people dying in 2022 [1]. One of the main strategies for ending this global epidemic is interrupting the chain of transmission by finding and treating all people with infectious forms of TB [2]. Several TB diagnostic tools have been introduced, including chest radiography, tuberculin skin test, interferon-gamma release assays, sputum smear microscopy, sputum mycobacterial culture, and various forms of nucleic acid amplification tests [3]. However, there are important challenges to implementing each of these diagnostic modalities where none of them is sufficient to stand alone in the diagnostic pathway. The most appropriate way to combine information from a range of sources remains unknown. For this reason, researchers have tried to incorporate various Artificial Intelligence (AI)-based algorithms to assist in the diagnosis and detection of TB disease.

ChatGPT

Tuberculosis (TB) stood as the predominant infectious disease threat globally before the onset of the COVID-19 pandemic, affecting over 10.5 million individuals and claiming the lives of 1.3 million people in 2022 [1]. A pivotal strategy in halting this global epidemic revolves around breaking the transmission chain by identifying and treating all individuals with infectious forms of TB [2]. Various diagnostic tools have been introduced to aid in this endeavor, including chest radiography, tuberculin skin tests, interferon-gamma release assays, sputum smear microscopy, sputum mycobacterial culture, and an array of nucleic acid amplification tests [3]. Nonetheless, each of these diagnostic modalities faces significant implementation challenges, and none alone is deemed adequate for the diagnostic pathway. The optimal approach for amalgamating information from diverse sources remains elusive. Consequently, researchers have endeavored to integrate Artificial Intelligence (AI)-based algorithms to augment the diagnosis and detection of TB disease.

user

improve this:

AI comprises any technique that enables computer programs to solve complex problems by mimicking human behaviour and thinking processes [4]. Recently, there are two popular terms commonly used to refer to AI techniques that require minimal to no human intervention, i.e., Machine Learning (ML) and Deep Learning (DL). Considered an integral part of AI, ML could find meaningful patterns in datasets automatically without being explicitly programmed [5]. Meanwhile, DL can be seen as a sub-field in ML that uses deep and complex neural networks to extract features or patterns in datasets for further analysis [5], [6].

ChatGPT

Artificial Intelligence (AI) encompasses a spectrum of techniques enabling computer programs to tackle intricate problems by emulating human behavior and cognitive processes [4]. Presently, two widely used terms denote AI

techniques that necessitate minimal to no human intervention: Machine Learning (ML) and Deep Learning (DL). Positioned as a fundamental component of AI, ML possesses the capability to discern meaningful patterns within datasets autonomously, without explicit programming [5]. Conversely, DL emerges as a subset within ML, leveraging deep and intricate neural networks to extract features or patterns from datasets for subsequent analysis [5], [6].

user

improve this:

There is an exponentially increasing trend of published articles related to TB detection using AI methods as depicted in Figure 1. This indicates the high potential and interest of researchers to use and develop AI methods for TB detection. However, it also confirms the need to comprehensively review published literature in this area to understand the diagnostic performance of AI-based methods for TB detection.

ChatGPT

The trend depicted in Figure 1 reveals an exponential rise in published articles concerning TB detection through AI methods. This trend underscores the considerable potential and interest among researchers in employing and advancing AI techniques for TB detection. Nevertheless, it also underscores the imperative to meticulously review existing literature in this domain to grasp the diagnostic efficacy of AI-based methods for TB detection.

user

improve this:

There are several related review papers in this domain. Jimmy et al [7] performed a systematic literature review on four databases with a focus on different DL methods for TB detection based on Chest X-Rays (CXR). Similarly, Sharma et al [8] also published a narrative survey on DL-based Convolutional Neural Networks (CNNs) models implemented for TB disease diagnosis from CXR. Zeyu et al [9] performed a narrative review to categorize and analyze different DL-based methods for TB diagnosis, and Oloko-Oba and Viriri [10] investigated state-of-the-art DL techniques for TB detection from chest radiographs. Santosh et al [11] also performed a systematic review with a focus on DL for TB screening using CXR but with a smaller time period of five years (2016-2021). Carvalho et al [12] published a systematic review with a focus on DL techniques used to classify TB bacilli in microscopic images of Ziehl-Neelsen (ZN) sputum smears. In contrast, Barros et al [13] conducted a systematic review that focused on ML models to predict TB treatment outcomes. All of these differ from our current review where our focus is not only on DL but also ML as integral part of AI-based methods for TB detection.

ChatGPT

In the realm of TB detection, several notable review papers have emerged. Jimmy et al [7] conducted a meticulous systematic literature review across four databases, delving into various DL methodologies for TB detection based on Chest X-Rays (CXR). Similarly, Sharma et al [8] contributed a narrative survey spotlighting DL-based Convolutional Neural Networks (CNNs) models tailored for TB disease diagnosis from CXR imagery. Zeyu et al [9]

embarked on a comprehensive narrative review aimed at categorizing and dissecting diverse DL-based approaches for TB diagnosis. Meanwhile, Oloko-Oba and Viriri [10] scrutinized cutting-edge DL techniques for TB detection via chest radiographs. Santosh et al [11] conducted a systematic review, albeit with a narrower timeframe spanning five years (2016-2021), focusing on DL for TB screening utilizing CXR. Carvalho et al [12] contributed a systematic review honing in on DL techniques employed for classifying TB bacilli in microscopic Ziehl-Neelsen (ZN) sputum smear images. Conversely, Barros et al [13] conducted a systematic review centering on ML models geared towards predicting TB treatment outcomes.

Distinguished from these endeavors, our current review takes a broader perspective, encompassing not only DL but also ML as integral components of AI-based methods for TB detection.

user

improve this:

Another review from Siddiqui and Garg [14] emphasized recent advancements in intelligent techniques, including ML and DL, for Pulmonary Tuberculosis (PTB) diagnosis. Meanwhile, Singh et al [15] focused on the limitations of conventional TB diagnostics and applications of both ML and DL methods for TB diagnosis. They also described some industrial-grade Computer Aided Detection (CAD) tools, such as CAD4TB, Lunit INSIGHT, qXR, and InferRead DR Chest, as prospective AI-tools in TB detection. Both differed from our review where we performed a systematic review to assess the diagnostic performance of ML and DL methods in TB detection.

ChatGPT

Siddiqui and Garg [14] delved into recent strides in intelligent techniques, encompassing both ML and DL, for diagnosing Pulmonary Tuberculosis (PTB). In a similar vein, Singh et al [15] scrutinized the drawbacks of conventional TB diagnostics while exploring the utility of ML and DL methodologies for TB diagnosis. Additionally, they highlighted several industrial-grade Computer-Aided Detection (CAD) tools, such as CAD4TB, Lunit INSIGHT, qXR, and InferRead DR Chest, as promising AI-driven instruments in TB detection. Notably, both of these reviews diverge from our own, wherein we undertook a systematic review aimed at evaluating the diagnostic efficacy of ML and DL methodologies in TB detection.

user

improve this:

Harris et al [16] conducted a systematic review focusing on the diagnostic accuracy of AI-based software for TB detection using CXR. Similarly, Hansun et al [17] recently published a systematic review that focuses on the efficacies of ML and DL methods for TB detection using CXR. Both are similar to our current review in terms of assessing the diagnostic performance of AI-based methods for TB detection. However, these reviews only included the studies that used CXR data type. In the current review, we included studies that used different types of data, namely, radiographic (e.g., CXR, Computed Tomography/CT, culture imaging, etc.), molecular (e.g., blood chemical data, plasma, etc.), and physiological (e.g., blood pressure, Body Mass Index/BMI, etc.).

ChatGPT

Harris et al [16] meticulously conducted a systematic review, centering on the diagnostic accuracy of AI-based software in TB detection utilizing CXR imagery. Similarly, Hansun et al [17] recently published a systematic review delving into the effectiveness of ML and DL methodologies for TB detection using CXR. Both studies align closely with our current review, as they also aim to evaluate the diagnostic performance of AI-based methods for TB detection. However, it's worth noting that these reviews solely encompass studies employing CXR data. In contrast, our review encompasses a broader spectrum, encompassing various data types such as radiographic (e.g., CXR, Computed Tomography/CT, culture imaging, etc.), molecular (e.g., blood chemical data, plasma, etc.), and physiological (e.g., blood pressure, Body Mass Index/BMI, etc.). This broader scope enables a more comprehensive evaluation of AI-based methods across diverse data modalities in TB detection.

user

improve this:

In the next section, we explain the applied method in conducting this systematic review, which is further divided into the following subsections: Design, Information Sources, Search Strategy, Inclusion and Exclusion Criteria, Data Extracted and Storage, Outcomes Assessed, Strategy for Data Analysis and Synthesis, and Publication Bias Assessment. In the Results section, we describe the General Characteristics of Included Studies, the Risk of Bias Assessment Results, and the review Study Results. In the Discussion section, first, we point out several Principal Findings, followed by Limitations and Conclusion of this systematic review.

ChatGPT

In the subsequent section, we delineate the methodological approach employed in conducting this systematic review, which is systematically structured into the following subsections: Design, Information Sources, Search Strategy, Inclusion and Exclusion Criteria, Data Extraction and Storage, Outcomes Assessed, Strategy for Data Analysis and Synthesis, and Assessment of Publication Bias.

Moving to the Results section, we provide a detailed overview of the General Characteristics of Included Studies, along with the Results of the Risk of Bias Assessment and the findings gleaned from the review Study Results.

Transitioning to the Discussion section, we initially highlight several Principal Findings gleaned from our analysis, followed by a critical appraisal of the Limitations encountered during the review process. Lastly, we conclude with a succinct summary encapsulating the key insights drawn from this systematic review.

user

improve this:

Diagnostic Performance of Artificial Intelligence-Based Methods for Tuberculosis Detection: A Systematic Review

ChatGPT

Enhancing the title:

"Evaluating the Diagnostic Accuracy of Artificial Intelligence-Based Methods for Tuberculosis Detection: A Comprehensive Systematic Review"

user

improve this:

In performing this systematic review, we followed the Preferred Reporting Items for Systematic Reviews and Meta-Analyses (PRISMA) 2020 Guidelines [18]. We prepared the review protocol based on the PRISMA-Protocol 2015 Statement [19], [20] which was then registered on the Prospective Register of Systematic Reviews (PROSPERO) to increase the transparency of the systematic review process [21]. The review protocol is available at PROSPERO with Record ID CRD42023453611 [22]. As this systematic review focuses on retrospective studies, no ethical approval was required.

ChatGPT

In conducting this systematic review, adherence to the Preferred Reporting Items for Systematic Reviews and Meta-Analyses (PRISMA) 2020 Guidelines [18] was paramount. Our review protocol, fashioned in accordance with the PRISMA-Protocol 2015 Statement [19], [20], underwent registration on the Prospective Register of Systematic Reviews (PROSPERO) to enhance the transparency of our methodology [21]. Interested parties can access the review protocol at PROSPERO under Record ID CRD42023453611 [22]. Given the retrospective nature of the studies included in this review, ethical approval was deemed unnecessary.

user

improve this:

There are three major academic search systems and online databases used as the information sources for this systematic review. They are Scopus, PubMed, and the Association for Computing Machinery (ACM) Digital Library. All three are recommended and principal sources for systematic reviews [23]. We traced all published literature available in those databases up to 25 July 2023 when this systematic review was started.

ChatGPT

The systematic review draws upon three primary academic search systems and online databases: Scopus, PubMed, and the Association for Computing Machinery (ACM) Digital Library. These platforms are widely recognized and esteemed as pivotal sources for comprehensive literature reviews [23]. To ensure thoroughness, our search encompassed all published literature within these databases up to July 25, 2023, marking the initiation of this systematic review.

user

improve this:

We used three main keywords for the search strategy, namely “Artificial Intelligence”, “Tuberculosis”, and “Detect*.” We also added several alternative terms for each main keyword to be considered in the searching process. Table 1 shows the main keywords together with their alternative terms that were proposed by SH and refined by AA.

ChatGPT

For our search strategy, we employed three primary keywords: "Artificial Intelligence," "Tuberculosis," and "Detect*." Additionally, we incorporated several alternative terms for each main keyword to ensure comprehensive coverage during the search process. Table 1 outlines the main keywords along with their respective alternative terms, initially proposed by SH and subsequently refined by AA.

user

Using the main keywords and alternative terms, we obtained results as shown in Table 2. A total of 1,146 records were found but some records were not downloadable from those databases directly. Therefore, we further searched not available records in a major search engine, viz. Google, to obtain as many records as possible according to the searched results. At last, we could download a total of 1,088 records. The 58 records which could not be accessed are listed in Appendix 1.

ChatGPT

Using the main keywords and alternative terms, we conducted searches resulting in the findings outlined in Table 2. A total of 1,146 records were identified; however, some records were inaccessible for direct download from the respective databases. Consequently, we undertook additional searches for the unavailable records utilizing a prominent search engine, namely Google, to maximize the retrieval of relevant records. Ultimately, we successfully downloaded a total of 1,088 records. The 58 records that remained inaccessible are enumerated in Appendix 1.

user

improve this:

Using the main keywords and alternative terms, we obtained results as shown in Table 2. A total of 1,146 records were found but some records were not downloadable from those databases directly. Therefore, we further searched not available records in a major search engine, viz. Google, to obtain as many records as possible according to the searched results. At last, we could download a total of 1,088 records. The 58 records which could not be accessed are listed in Appendix 1.

ChatGPT

Employing the main keywords and their alternative terms, we conducted searches resulting in the findings outlined in Table 2. Initially, we identified a total of 1,146 records; however, certain records were not directly downloadable from the respective databases. Consequently, we extended our search to inaccessible records using a prominent search engine, Google, to maximize the retrieval of relevant literature. Ultimately, we successfully obtained a total of 1,088 records. For

reference, the 58 records that remained inaccessible are listed in Appendix 1.

user

improve this:

Using the main keywords and alternative terms, we obtained results as shown in Table 2. A total of 1,146 records were found but some records were not downloadable from those databases directly. Therefore, we further searched not available records in a major search engine, viz. Google, to obtain as many records as possible according to the searched results. At last, we could download a total of 1,088 records. The 58 records which could not be accessed are listed in Appendix 1.

ChatGPT

Utilizing the main keywords and their corresponding alternative terms, we conducted searches that yielded the results depicted in Table 2. While a total of 1,146 records were initially identified, it was noted that certain records could not be directly downloaded from the respective databases. Consequently, we extended our search to include inaccessible records through a major search engine, specifically Google, in order to maximize the retrieval of relevant literature. Ultimately, we successfully retrieved and downloaded a total of 1,088 records. For completeness, the 58 records that remained inaccessible are detailed in Appendix 1.

user

improve this:

All downloaded records were then checked against the inclusion and exclusion criteria adopted in this review study as shown in Table 3. These criteria help us in deciding to include or reject records during each phase of the PRISMA 2020 Guidelines.

ChatGPT

Subsequently, all downloaded records underwent scrutiny against the inclusion and exclusion criteria outlined for this review study, as illustrated in Table 3. These predefined criteria served as the guiding framework for our decision-making process, enabling us to determine whether to include or exclude records at each phase in alignment with the PRISMA 2020 Guidelines.

user

improve this:

Information extracted from each included study includes records metadata (title, authors, published year, journal or proceeding’s title), studies’ objectives and findings, dataset characteristics (source and total number of data), modality, type of data, AI methods applied, evaluation technique used, performance metrics, best results, comparison with other studies, outcome types (model development, CAD tool, clinical evaluation), Google Scholar citation counts, and sponsors or funding sources.

ChatGPT

The information extracted from each included study encompasses a comprehensive array of data points, including metadata such as title, authors, publication year, journal or proceeding's title, alongside the objectives and findings of the studies. Furthermore, dataset characteristics, including their source and total number of data points, are documented, along with details regarding the modality and type of data utilized. The AI methods applied, evaluation techniques employed, performance metrics, best results obtained, and any comparisons with other studies are meticulously recorded. Additionally, outcome types, ranging from model development to Computer-Aided Detection (CAD) tool assessment and clinical evaluation, are cataloged. The citation counts from Google Scholar, as well as information regarding sponsors or funding sources, are also gathered as part of the comprehensive extraction process.

user

improve this:

All collected and downloaded records were stored in the University of New South Wales (UNSW) OneDrive storage platform. All review authors were involved in the concept development and all phases of PRISMA 2020 Guidelines (Identification, Screening, and Included) in their respective roles. We recorded and documented each phase's results using a standardized spreadsheet document and conducted regular online discussions among review authors.

ChatGPT

The entirety of collected and downloaded records were meticulously stored within the University of New South Wales (UNSW) OneDrive storage platform for safekeeping and accessibility. Each review author was actively engaged in the conceptualization and execution of all phases outlined in the PRISMA 2020 Guidelines, including Identification, Screening, and Inclusion, each fulfilling their designated roles. To ensure accuracy and transparency, we meticulously documented the results of each phase using a standardized spreadsheet document. Additionally, regular online discussions were conducted among review authors to facilitate collaboration and maintain coherence throughout the review process.

user

improve this:

The main outcome of this systematic review is the list of various AI methods used for TB detection. The list then can be used to prioritize popular AI methods available in the literature for TB detection based on different biomarker types.

ChatGPT

The primary outcome of this systematic review is the compilation of diverse AI methodologies employed for TB detection. This curated list serves as a valuable resource, enabling the prioritization of popular AI methods documented in the literature for TB detection across various biomarker types. Such prioritization facilitates informed decision-making and

supports the identification of optimal approaches tailored to specific diagnostic contexts.

user

improve this:

The secondary outcome is the summary of the diagnostic performance of various AI methods in TB detection, including accuracy, area under the ROC curve (AUC), sensitivity, and specificity.

ChatGPT

The secondary outcome encompasses a comprehensive summary of the diagnostic performance exhibited by various AI methods in TB detection. This summary encompasses key metrics such as accuracy, area under the ROC curve (AUC), sensitivity, and specificity, providing valuable insights into the efficacy and reliability of these methodologies in clinical practice.

user

improve this:

We used a systematic narrative synthesis in the text based on extracted information from included studies. We also performed descriptive statistics, mainly using the Box and Whisker plots along with other tables and figures to summarize and describe the characteristics and key findings of this systematic review. The narrative synthesis was used to explore the relationship and findings both within and between included studies differentiated by several attributes, such as modality, biomarker types, and AI methods. We did not perform a meta-analysis due to the heterogeneity in designs, comparators, biomarker types, evaluation, and AI methods included in this review study.

ChatGPT

We adopted a systematic narrative synthesis approach, integrating information extracted from the included studies within the text. Descriptive statistics, primarily employing Box and Whisker plots alongside other tables and figures, were utilized to encapsulate and elucidate the characteristics and pivotal findings unearthed in this systematic review. The narrative synthesis method facilitated an in-depth exploration of the relationships and insights both within and across included studies, stratified by various attributes such as modality, biomarker types, and AI methods. Given the pronounced heterogeneity observed in study designs, comparators, biomarker types, evaluation methodologies, and AI methods encompassed in this review study, a meta-analysis was deemed impractical.

user

improve this:

To assess the risk of bias within the included studies, we applied a modified Quality Assessment of Diagnostic Accuracy Studies version 2 (QUADAS-2) tool. It is a recommended tool to be used in systematic reviews to evaluate the risk of bias and applicability of primary diagnostic accuracy studies [24], with four key domains, namely 1) patient selection,

2) index test, 3) reference standard, and 4) flow and timing. Questions in the QUADAS-2 tool were modified to accommodate the purpose of this review in evaluating AI-based methods' performance in TB detection. SH and AW performed the assessment independently. In case of disagreements, it was resolved first by follow-up discussion between SH and AW, and then by asking for a third opinion (AA and/or GBM) if needed.

ChatGPT

In assessing the risk of bias within the included studies, we utilized a modified version of the Quality Assessment of Diagnostic Accuracy Studies version 2 (QUADAS-2) tool. This tool is widely recommended for systematic reviews as it enables the evaluation of bias and applicability in primary diagnostic accuracy studies [24]. It comprises four key domains: 1) patient selection, 2) index test, 3) reference standard, and 4) flow and timing. To tailor the assessment to the specific focus of this review on evaluating AI-based methods' performance in TB detection, adjustments were made to the questions in the QUADAS-2 tool. The assessment was carried out independently by SH and AW. In instances of discrepancies, resolution was initially attempted through follow-up discussions between SH and AW, with involvement from AA and/or GBM for a third opinion if necessary.

user

improve this:

Figure 2 illustrates all processes performed throughout the systematic review. We followed the PRISMA 2020 flowchart with some modifications by adding the process timeline and registered protocol status information in PROSPERO.

ChatGPT

Figure 2 delineates all the processes conducted throughout the systematic review. We adhered to the PRISMA 2020 flowchart with certain modifications, including the incorporation of a process timeline and the inclusion of registered protocol status information in PROSPERO.

user

improve this:

Out of 1,146 records identified from Scopus (n=857), PubMed (n=284), and ACM Digital Library (n=5), 58 were not available. We checked and removed duplicates (n=227), which leaves us with 861 records to be passed to the screening phase. All records' titles, abstracts, and keywords were examined using four main rejection reasons, namely 1) not written in English (n=2); 2) not full-text research paper (n=89); 3) not published, not reviewed, or retracted (n=2); and 4) not focused on AI methods for TB detection (n=355). After these exclusions, we have 413 records remaining for eligibility assessment.

ChatGPT

Out of the 1,146 records initially identified from Scopus (n=857), PubMed (n=284), and ACM Digital Library (n=5), 58 were found to be unavailable. Upon checking and removing duplicates (n=227), we were left with 861 unique

records to be passed to the screening phase. Subsequently, all records' titles, abstracts, and keywords underwent examination based on four primary rejection criteria: 1) not written in English (n=2); 2) not a full-text research paper (n=89); 3) not published, not reviewed, or retracted (n=2); and 4) not focused on AI methods for TB detection (n=355). Following these exclusions, 413 records remained for eligibility assessment.

user

improve this:

All 413 records were assessed by reading their full-text content. Out of 413, 261 articles were excluded for the following reasons: 1) have unclear datasets (n=64); 2) have unclear methodologies or method and evaluation (n=39); 3) have finding inconsistency (n=28); and 4) not focused on AI methods for TB detection (n=130). This leaves us with a total of 152 articles as included studies in this systematic review. We then performed the data extraction process for all included studies. The overall review process took around six months starting on 25 July 2023 (Identification) until 22 January 2024 (Included and data extraction).

ChatGPT

All 413 records underwent thorough assessment by examining their full-text content. From this pool, 261 articles were excluded due to the following reasons: 1) unclear datasets (n=64); 2) unclear methodologies or method and evaluation (n=39); 3) inconsistencies in findings (n=28); and 4) lack of focus on AI methods for TB detection (n=130). This resulted in a total of 152 articles being included as part of this systematic review. Subsequently, the data extraction process was carried out for all included studies. The entirety of the review process spanned approximately six months, commencing on July 25, 2023 (Identification), and concluding on January 22, 2024 (Inclusion and data extraction).

user

improve this:

A summary of the general characteristics of 152 included studies in this systematic review is depicted in Figure 3. Most of the included studies used a single modality (n=139) compared to a multimodal (n=13) approach. Radiographic become the most popularly used data type (n=129) than molecular (n=21) and physiologic (n=16) data types, noting that several studies that used a multimodal approach may use more than one data type, for example, radiographic together with physiologic data types.

ChatGPT

A summary of the general characteristics of the 152 included studies in this systematic review is illustrated in Figure 3. The majority of the included studies opted for a single modality (n=139), with a smaller subset employing a multimodal approach (n=13). Among the various data types utilized, radiographic data emerged as the most prevalent (n=129), followed by molecular (n=21) and physiologic (n=16) data types. It's noteworthy that several studies employing a multimodal approach integrated more than one data type, such as a combination of radiographic and physiologic data types.

user

improve this:

Regarding the evaluation technique, most studies (n=95) used the holdout evaluation technique that split the whole dataset into training and test sets. Forty-eight studies used the k-fold Cross Validation (CV) technique that divides the dataset into k partition and performs the evaluation k times. Only nine studies involved external datasets in the evaluation phase. 143 out of 152 included studies focused on the AI model development rather than application or prototype creation (n=8) and clinical evaluation of AI-based CAD tools (n=1).

ChatGPT

In terms of evaluation techniques, a majority of studies (n=95) employed the holdout evaluation technique, which involves dividing the entire dataset into training and test sets. Forty-eight studies utilized the k-fold Cross Validation (CV) technique, dividing the dataset into k partitions and conducting the evaluation k times. Only nine studies incorporated external datasets during the evaluation phase. Out of the 152 included studies, 143 focused on AI model development, while eight studies concentrated on application or prototype creation, and one study delved into the clinical evaluation of AI-based Computer-Aided Detection (CAD) tools.

user

improve this:

Around 89 studies used the Transfer Learning (TL) approach. TL is a relatively new and proven strategy for solving domain-specific problems [25], particularly in studies that used DL methods. Sixty-eight included studies compared their results with several other previous studies. An equal proportion between studies that got funded or sponsored by various entities (n=78) and studies that did not get any funding (n=74) was spotted. All included studies were published between 2011 to 2023.

ChatGPT

Approximately 89 studies employed the Transfer Learning (TL) approach, a relatively recent and validated strategy for addressing domain-specific challenges [25], especially prevalent in studies utilizing Deep Learning (DL) methods. Moreover, 68 included studies conducted comparisons with multiple prior studies. Notably, an equal proportion of studies received funding or sponsorship from various entities (n=78) compared to those that did not receive any funding (n=74). It's noteworthy that all included studies were published between 2011 and 2023.

user

improve this:

141 different data sources were identified from the included studies with a total of 2,082,457 records. The most used data sources were Shenzhen (SZ; n=51), Montgomery County (MC; n=46); Kaggle TB CXR (n=6), and TBX11K (n=5), all in the form of Radiographic data type. MC and SZ are clearly the

dominant data sources used in these studies. Both of them are publicly available CXR datasets from the U.S. National Library of Medicine with 138 images available in MC and 662 images in SZ [26]. Meanwhile, the Kaggle TB CXR dataset contains 7,000 images but only 4,200 of them are publicly accessible [27]. TBX11K is a newer and larger CXR dataset for TB-related studies. It was introduced in 2020 with a total of 11,200 images [28]. More detailed information regarding extracted data from all included studies is presented in Appendix 2.

ChatGPT

A total of 141 distinct data sources were identified across the included studies, comprising a vast collection of 2,082,457 records. Among these, the most frequently utilized data sources included Shenzhen (SZ; n=51), Montgomery County (MC; n=46), Kaggle TB CXR (n=6), and TBX11K (n=5), all of which provided Radiographic data. Notably, MC and SZ emerged as the predominant data sources, both publicly available CXR datasets from the U.S. National Library of Medicine. MC contains 138 images, while SZ offers 662 images [26]. The Kaggle TB CXR dataset boasts 7,000 images, though only 4,200 are publicly accessible [27]. Conversely, TBX11K, introduced in 2020, stands out as a newer and more extensive CXR dataset for TB-related research, featuring a total of 11,200 images [28]. For further insights into the extracted data from all included studies, detailed information is available in Appendix 2.

user

improve this:

Figure 4 illustrates the QUADAS-2 assessment results, specifically for the risk of bias and applicability concerns for all included studies. There were 18 studies [29]–[46] with a high risk of bias and one study [47] with unclear risk in terms of "patient selection." This is mainly due to incomplete or missing information in the data selection process. Four studies [48]–[51] were identified to have high risk in terms of "index test" due to missing information on model architecture and parameters used in those studies. Three studies [42], [52], [53] were grouped as having high risk, and eight studies [31], [33], [43], [44], [54]–[57] as unclear risk in terms of "reference standard." This is because of incomplete or missing information about reference standards used in those studies. In terms of "flow and timing," two studies [45], [58] were categorized as having a high risk of bias, and eight studies [47], [52], [59]–[64] were assessed with unclear risk. This is mainly because of unclear or missing information regarding the time interval and intervention given in those studies.

ChatGPT

Figure 4 presents the QUADAS-2 assessment outcomes, specifically highlighting the risk of bias and applicability concerns across all included studies. Among the assessed studies [29]–[46], 18 were flagged for a high risk of bias, with one study [47] marked as having an unclear risk concerning "patient selection," largely attributable to incomplete or absent information in the data selection process. Moreover, four studies [48]–[51] were identified as having a high risk regarding "index test," attributed to the absence of crucial details on model architecture and parameters. Additionally, three studies [42], [52], [53] were classified as having a high risk, and eight studies [31], [33], [43], [44], [54]–[57] as unclear risk concerning "reference standard," due to incomplete or missing

information regarding the reference standards employed. Concerning "flow and timing," two studies [45], [58] were deemed to have a high risk of bias, while eight studies [47], [52], [59]-[64] were assigned an unclear risk, primarily due to unclear or absent information regarding the time interval and intervention provided in those studies.

user

improve this:

Regarding the applicability concerns, most of the included studies had low concerns indicating their proposed solutions are feasible and applicable. There were only three studies [32], [40], [58] with high risk and one study [47] with unclear risk in terms of "patient selection", three studies [48], [51], [65] with high risk in terms of "index test", one study [53] with high risk and eight studies [31], [33], [43], [44], [54]-[57] with unclear risk in terms of "reference standard". The full QUADAS-2 assessment results are provided in Appendix 3.

ChatGPT

Concerning applicability concerns, the majority of the included studies exhibited low concerns, suggesting the feasibility and practical applicability of their proposed solutions. Only three studies [32], [40], [58] were identified with a high risk, and one study [47] with an unclear risk in terms of "patient selection." Additionally, three studies [48], [51], [65] were flagged with a high risk concerning "index test," while one study [53] showed a high risk, and eight studies [31], [33], [43], [44], [54]-[57] had an unclear risk concerning "reference standard." For comprehensive QUADAS-2 assessment results, please refer to Appendix 3.

user

improve this:

Various AI-based algorithms have been introduced and used in the included studies. These can be grouped into two major approaches, namely ML and DL. 102 studies utilized DL [28]-[31], [33]-[35], [39], [40], [42], [45], [48], [50], [51], [55]-[62], [64], [66]-[144], 30 employed ML [32], [36], [41], [43], [44], [47], [52]-[54], [65], [145]-[164], and 19 used both ML and DL [37], [38], [46], [49], [63], [165]-[178]. One study [179] focused on the DL-based CAD tool, viz. Lunit INSIGHT v4.7.2, for systematic screening of TB in a low-prevalence setting.

ChatGPT

The included studies have implemented various AI-based algorithms, which can be broadly categorized into two major approaches: Machine Learning (ML) and Deep Learning (DL). Specifically, 102 studies utilized DL methods [28]-[31], [33]-[35], [39], [40], [42], [45], [48], [50], [51], [55]-[62], [64], [66]-[144], while 30 studies employed ML techniques [32], [36], [41], [43], [44], [47], [52]-[54], [65], [145]-[164]. Additionally, 19 studies employed a combination of both ML and DL approaches [37], [38], [46], [49], [63], [165]-[178]. Furthermore, one study [179] focused on the implementation of a DL-based Computer-Aided Detection (CAD) tool, namely Lunit INSIGHT v4.7.2, specifically designed for systematic TB screening in a low-prevalence setting.

user

improve this:

Figure 5 depicts different ML methods used in the included studies. Among the different kinds of ML methods used, Support Vector Machine (SVM), Random Forest (RF), and Logistic Regression (LR), were the three most popular ones. More specifically, 33 studies employed SVM, 17 applied RF, and 15 used LR.

ChatGPT

Figure 5 illustrates the diverse array of Machine Learning (ML) methods utilized in the included studies. Notably, Support Vector Machine (SVM), Random Forest (RF), and Logistic Regression (LR) emerged as the three most prevalent ML techniques. Specifically, 33 studies employed SVM, 17 utilized RF, and 15 applied LR as part of their methodologies.

user

improve this:

For DL, Convolutional Neural Networks (CNNs) have undoubtedly become the dominantly used method among included studies in this systematic review. Despite the custom CNNs (n=39), many researchers utilized well-known DL architectures, including VGG-16 (n=37), ResNet-50 (n=33), and DenseNet-121 (n=19). Figure 6 summarizes different DL methods found in the included studies.

ChatGPT

In the realm of Deep Learning (DL), Convolutional Neural Networks (CNNs) have unequivocally emerged as the predominant method among the included studies in this systematic review. Alongside custom CNNs (n=39), many researchers have leveraged established DL architectures, notably including VGG-16 (n=37), ResNet-50 (n=33), and DenseNet-121 (n=19). Figure 6 provides a comprehensive overview of the various DL methods identified in the included studies.

user

improve this:

The most used performance evaluation metrics were accuracy (n=128), sensitivity (n=112), area under the ROC curve (AUC) (n=87), and specificity (n=78). Accuracy is the proportion of correctly predicted cases, either true positives or true negatives, with all cases [180]. Sensitivity, also known as recall or True Positive Rate (TPR), is defined as a model’s ability to correctly identify people with a condition [181], [182]. AUC is derived from the Receiver Operating Characteristics (ROC) curve to quantitatively measure a model’s performance [180]. Specificity or True Negative Rate (TNR) is the model’s ability to correctly identify people without a condition [181], [182]. Figure 7 shows the proportion of various performance metrics found in the included studies. A detailed description of performance metrics used in the included studies is presented in Appendix 4.

ChatGPT

The most frequently employed performance evaluation metrics encompassed accuracy (n=128), sensitivity (n=112), area under the ROC curve (AUC) (n=87), and specificity (n=78). Accuracy denotes the proportion of correctly predicted cases, encompassing true positives and true negatives among all cases [180]. Sensitivity, also recognized as recall or True Positive Rate (TPR), measures a model's ability to accurately identify individuals with a condition [181], [182]. AUC, derived from the Receiver Operating Characteristics (ROC) curve, quantitatively assesses a model's performance [180]. Specificity, or True Negative Rate (TNR), gauges a model's accuracy in identifying individuals without a condition [181], [182]. Figure 7 visually presents the distribution of various performance metrics identified in the included studies. For an in-depth exploration of the performance metrics employed across the included studies, refer to the detailed description provided in Appendix 4.

user

improve this:

Figure 8 shows the overall performance results in terms of accuracy, AUC, sensitivity, and specificity of all included studies. As can be inferred from the Box plot, accuracy ranged from 46.48% [68] to 100% [130] with a mean value of 91.93% and median of 93.59%; AUC ranged from 45.96% [68] to 100% [46], [95], [101], [115] with a mean value of 93.48% and median of 95.28%; sensitivity ranged from 60% [52] to 100% [46], [47], [70], [107], [117], [130], [137], [141], [156], [174], [179] with a mean value of 92.63% and median of 93.75%; and specificity ranged from 53.2% [138] to 100% [49], [126], [149], [178] with a mean value of 92.46% and median of 95.4%. All metrics are negatively skewed and have relatively the same distribution. There are some outliers detected for each metric, four for accuracy at 46.48% [68], 63% [136], 63.9% [138], and 70% [52]; two for AUC at 45.96% [68] and 69.2% [138]; three for sensitivity at 60% [52], 65.52% [164], and 69.07% [154]; and five for specificity at 53.2% [138], 60% [156], 60.8% [171], 69.44% [61], and 73% [162] of the included studies.

ChatGPT

Figure 8 provides a comprehensive overview of the overall performance results in terms of accuracy, AUC, sensitivity, and specificity across all included studies. From the depicted Box plot, it is evident that accuracy ranged from 46.48% [68] to 100% [130], with a mean value of 91.93% and a median of 93.59%. Similarly, AUC ranged from 45.96% [68] to 100% [46], [95], [101], [115], with a mean value of 93.48% and a median of 95.28%. Sensitivity spanned from 60% [52] to 100% [46], [47], [70], [107], [117], [130], [137], [141], [156], [174], [179], with a mean value of 92.63% and a median of 93.75%. Meanwhile, specificity ranged from 53.2% [138] to 100% [49], [126], [149], [178], with a mean value of 92.46% and a median of 95.4%. Notably, all metrics exhibited negative skewness and displayed relatively similar distributions. Furthermore, several outliers were detected for each metric, including four for accuracy at 46.48% [68], 63% [136], 63.9% [138], and 70% [52]; two for AUC at 45.96% [68] and 69.2% [138]; three for sensitivity at 60% [52], 65.52% [164], and 69.07% [154]; and five for specificity at 53.2% [138], 60% [156], 60.8% [171], 69.44% [61], and 73% [162] of the included studies.

user

improve this:

We further compared the performance based on several other criteria, such as whether the study applied the Transfer Learning (TL) method or not and whether the study used a single or multimodal approach. Figure 9 shows the split-grouped violin plots for comparative performance results of included studies based on TL and modality. A violin plot combines information we can get from a Box plot with a kernel density plot that enables us to visualize the distribution or density of numerical data.

ChatGPT

We conducted further performance comparisons based on several criteria, including whether the study employed the Transfer Learning (TL) method and whether it adopted a single or multimodal approach. Figure 9 presents split-grouped violin plots illustrating the comparative performance results of the included studies based on TL and modality. A violin plot amalgamates information from a Box plot with a kernel density plot, providing a visual depiction of the distribution or density of numerical data.

user

improve this:

We also compared the performance of AI methods used in all the included studies based on the AI methods applied (ML, DL, or both ML/DL) and data or biomarker types used (Radiographic, Molecular, Physiologic). Figure 10 illustrates the Box plots for comparative performance results of the included studies based on AI methods and biomarker types.

ChatGPT

We further examined the performance of AI methods utilized in all the included studies based on the AI techniques applied (ML, DL, or both ML/DL) and the types of data or biomarkers used (Radiographic, Molecular, Physiologic). Figure 10 showcases the Box plots, offering a comparative analysis of the performance results of the included studies based on AI methods and biomarker types.

user

improve this:

We systematically investigated available evidence related to the usage and diagnostic performance of various AI-based methods for TB detection. Among 152 included studies, the majority of them were published in the last eight years with only three studies published before 2016 [44], [145], [161]. From the included studies, 6,070 Google Scholar citations were recorded by January 18, 2024. The top three cited publications were Lakhani and Sundaram [110] with 1,695 citations, Pasa et al [121] with 312 citations, and Lopes and Valiati [168] with 260 citations. This confirms the increasing interest of researchers to incorporate AI-based methods for TB detection studies.

ChatGPT

We conducted a systematic exploration of available evidence concerning the utilization and diagnostic efficacy of various AI-based methods for TB detection. Among the 152 included studies, the majority were published within the last eight years, with only three studies predating 2016 [44], [145], [161]. As of January 18, 2024, these studies collectively garnered 6,070 citations on Google Scholar. Remarkably, the top three cited publications were identified as Lakhani and Sundaram [110] with 1,695 citations, Pasa et al. [121] with 312 citations, and Lopes and Valiati [168] with 260 citations. This underscores the burgeoning interest among researchers in integrating AI-based methodologies into TB detection studies.

user

improve this:

Different forms of data types were found which are grouped into radiologic, molecular, and physiologic. Although some recent studies used complex data types, such as long non-coding Ribonucleic Acid (RNAs) [158], [162] and exhaled Volatile Organic Compounds (VOCs) from human breath [156], [160], radiographic data is still dominantly used for TB detection studies. Overall, around 84.87% (n=129) of the included studies used radiographic data type, especially CXRs. Moreover, SZ and MC become the most used datasets with 51 and 46 of the included studies used them respectively. In total, there are 800 CXRs available in SZ and MC which is considered very small to achieve reasonable performance results, particularly for studies that employ DL methods. Therefore, many techniques were introduced to increase the data quantity [183], [184], including conventional data augmentation by using geometric transformations, random erasing, image mixing, color and feature space transformations [34], [74], [94], [95], [105], [110], [132], and even DL-based augmentation techniques, such as variational autoencoders and Generative Adversarial Network (GAN) [71]. However, proper data curation is undoubtedly needed to make sure the application of these augmented data could achieve the intended results for TB detection studies.

ChatGPT

Various forms of data types were identified, classified into radiologic, molecular, and physiologic categories. While recent studies have explored complex data types such as long non-coding Ribonucleic Acid (RNAs) [158], [162] and exhaled Volatile Organic Compounds (VOCs) from human breath [156], [160], radiographic data remains predominant in TB detection research. Approximately 84.87% (n=129) of the included studies utilized radiographic data types, particularly Chest X-Rays (CXRs). Notably, the SZ and MC datasets emerged as the most frequently utilized, with 51 and 46 studies respectively making use of them. However, it's noteworthy that the availability of data in SZ and MC, totaling 800 CXRs, is relatively limited, posing challenges, especially for studies employing Deep Learning (DL) methods where larger datasets are often required for optimal performance. Consequently, numerous techniques have been introduced to augment data quantity [183], [184], including conventional methods like geometric transformations, random erasing, image mixing, and color and feature space transformations [34], [74], [94], [95], [105], [110], [132], as well as DL-based augmentation techniques such as variational autoencoders and Generative Adversarial Network (GAN) [71]. However, it is crucial to ensure proper data curation to ensure that the application of these augmented data yields the desired outcomes for TB detection studies.

user

improve this:

On the other hand, around 13.82% (n=21) and 10.52% (n=16) used molecular and radiographic data types respectively. Among studies that used molecular data type [47], [49], [52], [53], [65], [102], [138], [146], [148], [150], [152]-[156], [158], [160], [162], [171], [174], [177], the highest accuracy, AUC, sensitivity, and specificity achieved were 99.2% [47], 99% [146], 100% [47], [156], [174], 100% [49] and the lowest were 63.9% [138], 69.2% [138], 60% [52], 53.2% [138]. Among studies that used physiologic data type [32], [36], [45], [52], [55], [56], [102], [108], [146], [147], [153], [158], [162], [171], [176], [177], the highest accuracy, AUC, sensitivity, and specificity achieved were 98% [146], 99% [146], 98% [146], 96.96% [177] and the lowest were 70% [52], 80.9% [171], 60% [52], 60.8% [171].

ChatGPT

Conversely, a smaller proportion of studies, approximately 13.82% (n=21), utilized molecular data types, while 10.52% (n=16) focused on physiologic data. Among those employing molecular data [47], [49], [52], [53], [65], [102], [138], [146], [148], [150], [152]-[156], [158], [160], [162], [171], [174], [177], the highest reported accuracy, AUC, sensitivity, and specificity were 99.2% [47], 99% [146], 100% [47], [156], [174], 100% [49], and the lowest were 63.9% [138], 69.2% [138], 60% [52], 53.2% [138]. Conversely, in studies utilizing physiologic data [32], [36], [45], [52], [55], [56], [102], [108], [146], [147], [153], [158], [162], [171], [176], [177], the highest reported accuracy, AUC, sensitivity, and specificity were 98% [146], 99% [146], 98% [146], 96.96% [177], while the lowest were 70% [52], 80.9% [171], 60% [52], 60.8% [171].

user

improve this:

Regarding the modality, most of the included studies used a single modality (one input data type, n=139) rather than a multimodal (multiple input data types, n=13) approach. As can be seen from the split-grouped violin plot in Figure 9 (right), studies that used single modality tend to have higher median and smaller distribution range than those that used multimodal approach, indicating the single modality approach could perform better than multimodal in terms of accuracy, AUC, sensitivity, and specificity. However, it is important to note that more outliers were detected in all performance metrics for the single modality than in the multimodal approach. It is also worth noting that these results were not applied under the same scenario setting. In general, when applied under the same scenario setting, the multimodal approach could enhance the performance results than the single modality [7], [185]. This as reported in some included studies that compare the single modality and multimodal results, such as Heo et al [102], Gozes and Greenspan [108], and Jimmy et al [45].

ChatGPT

Concerning modality, the majority of included studies adopted a single modality (one input data type, n=139) rather than employing a multimodal approach (utilizing multiple input data types, n=13). As illustrated in the split-grouped violin plot in Figure 9 (right), studies utilizing a single

modality tended to exhibit higher median values and narrower distribution ranges compared to those employing a multimodal approach. This suggests that the single modality approach may outperform the multimodal approach in terms of accuracy, AUC, sensitivity, and specificity. However, it's noteworthy that more outliers were observed in all performance metrics for the single modality compared to the multimodal approach. Additionally, it's important to acknowledge that these outcomes were not obtained under identical scenario settings. Generally, when applied under similar scenario settings, the multimodal approach has demonstrated the potential to enhance performance results compared to the single modality [7], [185]. This is supported by findings from several included studies that directly compared single modality and multimodal approaches, such as Heo et al. [102], Gozes and Greenspan [108], and Jimmy et al. [45].

user

improve this:

Most of the included studies (~94%) also focused on model development and refinement to achieve better and more accurate performance results for TB detection tasks rather than creating or deploying the model in the form of a working application or prototype. Meanwhile, as we can see from the performance metrics summary, all methods obtained very high mean and median performance metrics, mainly above 90%, indicating the conformity and maturity of the application of AI-based methods for TB detection. Therefore, there is a need to expand the focus of future studies to not only develop and refine the model performance but also to build and evaluate the model prototype as a proposed solution.

ChatGPT

The vast majority of included studies (~94%) primarily concentrated on the development and fine-tuning of models to achieve heightened accuracy and performance in TB detection tasks, rather than focusing on deploying the model as a functional application or prototype. Moreover, upon examining the summary of performance metrics, it becomes evident that all methods achieved remarkably high mean and median performance metrics, typically surpassing 90%. This suggests a high level of consistency and maturity in the application of AI-based methods for TB detection. Consequently, there arises a necessity to broaden the scope of future studies beyond mere model development and refinement, encompassing the creation and evaluation of model prototypes as viable solutions.

user

improve this:

Regarding the evaluation techniques used in the included studies, the holdout technique was dominantly used over k-fold cross-validation and external dataset validation. For medical use cases such as TB disease detection, it is recommended to use a more reliable evaluation technique than the holdout technique, which simply splits the dataset into training, validation, and test sets. Some of the included studies even used only one iteration of training and test sets without any validation set [29], [33], [35], [36], [41], [44], [46], [52], [53], [57], [63], [66], [68], [71], [72], [74], [80]-[82], [88], [93], [94], [107], [109], [111], [113], [114], [116], [128], [129], [131]-[133], [135], [136], [144]-[146], [149], [152], [153], [159]-[163].

ChatGPT

In the evaluation techniques employed across the included studies, the holdout technique emerged as the predominant choice, overshadowing k-fold cross-validation and external dataset validation. However, for critical medical applications like TB disease detection, it's advisable to opt for more robust evaluation methods than the holdout technique. The holdout technique, which essentially partitions the dataset into training, validation, and test sets, may lack the reliability required for medical scenarios. Notably, some studies even utilized a single iteration of training and test sets without incorporating a validation set. This practice, observed in numerous included studies, could potentially compromise the rigor and validity of the evaluation process [29], [33], [35], [36], [41], [44], [46], [52], [53], [57], [63], [66], [68], [71], [72], [74], [80]–[82], [88], [93], [94], [107], [109], [111], [113], [114], [116], [128], [129], [131]–[133], [135], [136], [144]–[146], [149], [152], [153], [159]–[163].

user

improve this:

DL has been used in a majority of included studies (n=102), where CNNs with VGG-16 (n=37), ResNet-50 (n=33), and DenseNet-121 (n=19) become the most popularly applied architectures. VGG-16 is a special type of VGGNet that has 16 weighted layers (13 convolutional layers and three fully connected layers) in the network's architecture. It was introduced by a group from Oxford University known as the Visual Geometry Group which took part and became the runner-up in ILSVRC 2014 [186]. The Residual Network or ResNet was introduced by He et al. [187] in 2015. It won first place on the ILSVRC 2015 classification task. ResNet-50 uses 50 weighted layers in the network which was deepest model at that time. Although it has a relatively high number of layers, ResNet-50 can be trained quickly due to the residual or skip connections used in the network [188]. Lastly, the DenseNet-121 is one of the most popular CNN architectures available in the literature. It was first introduced in a 2017 seminal paper by Huang et al. [189]. It densely connects all layers in the networks where each layer receives collective knowledge from all preceding layers and achieves cutting-edge results in image classification tasks.

ChatGPT

DL has emerged as a cornerstone in the majority of the included studies (n=102), with CNNs taking the forefront. Among these, architectures such as VGG-16, ResNet-50, and DenseNet-121 have gained notable traction. VGG-16, an evolution of the VGGNet series, boasts a distinctive architecture featuring 16 weighted layers, comprising 13 convolutional layers and three fully connected layers. Originating from the Visual Geometry Group at Oxford University, VGG-16 achieved runner-up status in the ILSVRC 2014 competition [186].

ResNet, introduced by He et al. in 2015, revolutionized deep learning with its residual connections. ResNet-50, a variant of ResNet, boasts 50 layers, marking it as one of the deepest models of its time. Despite its complexity, ResNet-50 offers efficient training owing to its ingenious use of residual or skip connections [187, 188].

DenseNet-121, another influential architecture, was introduced by Huang et al. in 2017. Renowned for its dense connectivity pattern, DenseNet-121

revolutionized information flow within CNNs by enabling each layer to receive direct input from all preceding layers. This approach enhances feature propagation and facilitates superior performance in image classification tasks [189].

user

improve this:

Other more recent DL architectures, such as EfficientNets, have also been utilized with promising results for TB detection. It was first introduced by Tan and Le in 2019 with eight variants, starting from EfficientNet-B0 up to EfficientNet-B7 [190]. Some included studies [29], [34], [39], [45], [59], [63], [72], [103], [104], [112], [115], [119], [120], [130], [131], [139], [140], [142], [143], [172], employed different variants of EfficientNets for TB detection, with EfficientNet-B0 emerging as the most commonly utilized variant (n=10). Some other studies [115], [130], [140] incorporated Vision Transformer (ViT), a transformer-like model specifically built for image recognition tasks. It was first introduced by Dosovitskiy et al [191] at the 2021 International Conference on Learning Representations (ICLR) and was well received in the computer vision community. Liu et al [192] also introduced a new family of ConvNet models, dubbed as ConvNeXts, to compete with ViT in image recognition tasks. This can be an interesting and promising model to be applied in medical image processing field, and especially the TB detection task.

ChatGPT

More recent DL architectures, including EfficientNets, have shown promise in TB detection. EfficientNets, introduced by Tan and Le in 2019, offer a range of variants from EfficientNet-B0 to EfficientNet-B7 [190]. Several studies [29], [34], [39], [45], [59], [63], [72], [103], [104], [112], [115], [119], [120], [130], [131], [139], [140], [142], [143], [172] have leveraged different variants of EfficientNets for TB detection, with EfficientNet-B0 being the most commonly utilized (n=10).

In addition, some studies have integrated Vision Transformer (ViT), a transformer-based model tailored for image recognition tasks. Initially proposed by Dosovitskiy et al. [191] at the 2021 International Conference on Learning Representations (ICLR), ViT garnered attention in the computer vision community. Furthermore, Liu et al. [192] introduced ConvNeXts, a family of ConvNet models designed to compete with ViT in image recognition tasks. This emerging model shows potential for application in medical image processing, particularly in TB detection tasks.

user

improve this:

Some included studies also experimented with various eXplainable AI (XAI) methods to enhance the trustworthiness of their AI models' outcomes. Originally developed to achieve a balanced tradeoff between interpretability and accuracy in machine learning (ML) and deep learning (DL) models [193], XAI has gained significant popularity. Several XAI techniques utilized in the included studies include Gradient-weighted Class Activation Mapping (Grad-CAM) [34], [62], [65], [79], [98], [103], [119], [126], [127], [140], [142], [143], Local Interpretable Model-agnostic Explanation (LIME) [127], and SHapley Additive exPlanations (SHAP) [127].

All of these techniques focus on visualizing the classification output from ML/DL models.

ChatGPT

Some of the included studies explored various eXplainable AI (XAI) methods to bolster the interpretability and trustworthiness of their AI models' outcomes. Originating from the need to strike a balance between interpretability and accuracy in machine learning (ML) and deep learning (DL) models [193], XAI has garnered considerable attention.

Among the XAI techniques utilized in the included studies are Gradient-weighted Class Activation Mapping (Grad-CAM) [34], [62], [65], [79], [98], [103], [119], [126], [127], [140], [142], [143], Local Interpretable Model-agnostic Explanation (LIME) [127], and SHapley Additive exPlanations (SHAP) [127]. These techniques are geared towards visualizing the classification output from ML/DL models, thereby enhancing their interpretability and transparency.

user

improve this:

Transfer Learning (TL) is a technique used to leverage knowledge gained from a source dataset and apply it to a target domain, relaxing the assumption that training data should be independent and identically distributed with test data [194]. TL is particularly useful in managing insufficient data volume when using DL methods.. Undoubtedly, TL has been widely employed in the studies included in this systematic review, especially those utilizing DL techniques, as evidenced by the application of various pre-trained DL models yielding promising results. Furthermore, as depicted in Figure 9 (left), studies implementing TL tend to exhibit higher median accuracy compared to others. They also demonstrate a narrower distribution range, indicating that TL-based approaches consistently yield higher and more consistent results in terms of accuracy, AUC, sensitivity, and specificity.

ChatGPT

Transfer Learning (TL) is a powerful technique that capitalizes on knowledge acquired from a source dataset and adapts it to a target domain, relaxing the assumption that training data must be independent and identically distributed with test data [194]. TL proves especially beneficial in scenarios with limited data volume, commonly encountered in Deep Learning (DL) applications.

Indeed, TL has been extensively utilized in the studies encompassed in this systematic review, particularly those employing DL methodologies. These studies have leveraged various pre-trained DL models, resulting in promising outcomes. Notably, as illustrated in Figure 9 (left), investigations integrating TL tend to exhibit higher median accuracy compared to counterparts. Additionally, they showcase a narrower distribution range, suggesting that TL-based approaches consistently deliver superior and more consistent results in terms of accuracy, Area Under the Curve (AUC), sensitivity, and specificity.

user

improve this:

In terms of the performance results, AI methods could achieve high accuracies with a mean value of 91.93% (median 93.59%), AUCs with a mean value of 93.48% (95.28%), sensitivities with a mean value of 92.63% (93.75%), and specificities with a mean value of 92.46% (95.4%). We further compared the performance results based on the AI methods used in the included studies, namely ML, DL, and both ML/DL. As can be seen in Figure 10 (left), DL has a higher accuracy with smaller variation than ML and ML/DL. However, in terms of AUC, sensitivity, and specificity, studies that applied ML/DL tend to have better results, followed by DL, and ML. This indicates that the combination of ML and DL may improve the model performance, however, very few studies have used it.

ChatGPT

In terms of performance metrics, the AI methods displayed remarkable achievements, with high accuracies averaging at 91.93% (median 93.59%), AUCs at 93.48% (median 95.28%), sensitivities at 92.63% (median 93.75%), and specificities at 92.46% (median 95.4%).

Further analysis based on the AI methods employed in the included studies, namely Machine Learning (ML), Deep Learning (DL), and the combination of both (ML/DL), reveals interesting insights. As illustrated in Figure 10 (left), DL exhibits higher accuracy with less variation compared to ML and ML/DL. However, concerning AUC, sensitivity, and specificity, studies integrating ML/DL tend to yield superior results, followed by DL, and then ML. This suggests that the fusion of ML and DL methodologies may enhance model performance, albeit few studies have explored this avenue.

user

improve this:

Related to the data types used in the included studies, we also compared the obtained performance results based on the biomarkers (data types), viz. radiographic, molecular, and physiologic. As can be seen in Figure 10 (right), studies that used radiographic data type achieved higher median scores in all performance metrics. It also has a better level of agreement than molecular and physiologic data types as indicated by the shorter interquartile ranges it has for all performance metrics. It means that studies with radiographic data types obtained more stable and consistent results.

ChatGPT

In addition to analyzing AI methods, we examined the performance results concerning the types of data used, specifically radiographic, molecular, and physiologic biomarkers. Figure 10 (right) illustrates the comparison.

Radiographic data type studies showcased superior median scores across all performance metrics. Notably, they demonstrated a higher level of consistency, evidenced by their shorter interquartile ranges in all performance metrics. This suggests that studies utilizing radiographic data types attained more stable and reliable outcomes compared to those using molecular and physiologic data types.

user

improve this:

In this systematic review, we also assessed the bias and applicability of all included studies using the QUADAS-2 tool. For the risk of bias, most of the included studies had low risks. However, among the four key aspects assessed, the patient selection aspect has the highest number of studies with high risk (n=18), meanwhile, the reference standard and flow and timing aspects both have the highest number of studies with unclear risk (n=8 for each aspect). Therefore, future studies should pay more attention to these aspects because they could affect our confidence in the published results of the studies. For the applicability concern, a majority of the included studies also had low concerns. This means that the proposed solutions in the included studies are feasible and applicable to solve the problem intended, i.e., TB disease detection.

ChatGPT

In our systematic review, we conducted an assessment of bias and applicability across all included studies utilizing the QUADAS-2 tool. Overall, most studies demonstrated low risks of bias.

However, when examining the four key aspects, we found that patient selection had the highest number of studies with a high risk (n=18). Additionally, both the reference standard and flow and timing aspects exhibited the highest number of studies with an unclear risk (n=8 for each aspect). These findings emphasize the importance of addressing these aspects in future research endeavors, as they could impact our confidence in the published results.

On the other hand, concerning applicability concerns, the majority of included studies demonstrated low concerns. This indicates that the proposed solutions presented in these studies are feasible and applicable for addressing the intended problem, specifically TB disease detection.

user

improve this:

Lastly, we also found that most of the included studies did not consider the evaluation of the proposed solutions to the domain-shift problem. There is only one included study [136] that conducted the domain-shift analysis in the TB detection task. As confirmed from this systematic review, AI-based methods have high performances in TB detection tasks using various kinds of data types. However, they were evaluated only on in-domain datasets where the evaluated data (validation and test sets) have the same data distribution and characteristics as the training data. It is known that most AI, particularly DL, methods have poor performances when applied to a real-world setting [195]. This may happen due to the shifted or different data distribution used during model development compared to the real-world dataset. Therefore, it is recommended to shift the focus to include, not only in-domain but also domain-shift analysis in future research.

ChatGPT

Finally, it's worth noting that the majority of the included studies overlooked the evaluation of proposed solutions regarding the domain-shift

problem. Remarkably, only one study [136] in our systematic review addressed domain-shift analysis in the context of TB detection.

While our review confirmed the high performance of AI-based methods across various data types in TB detection tasks, these evaluations were predominantly conducted on in-domain datasets. In other words, the datasets used for validation and testing shared the same distribution and characteristics as the training data. However, it's recognized that many AI methods, particularly deep learning approaches, struggle when applied to real-world settings [195]. This discrepancy often arises due to differences in data distribution between the training data and real-world datasets.

As such, it's imperative to broaden our focus to include not only in-domain evaluations but also domain-shift analysis in future research endeavors. By doing so, we can better understand and mitigate the challenges associated with deploying AI solutions in real-world scenarios.

user

improve this:

There are some limitations to this systematic review. Firstly, we only used three major academic search systems and online databases, namely Scopus, PubMed, and ACM Digital Library, as information sources. We believe that these databases adequately represent all studies within the domain of AI-based methods for TB detection. Secondly, we did not seek further information regarding three key aspects with high and unclear risks from the publication bias assessment. As most included studies used radiographic datasets, especially in the form of publicly available CXRs, the patient selection, reference standard, and flow and timing were assumed to be the same. Lastly, we did not perform a meta-analysis due to the heterogeneity in studies' designs, comparators, biomarker types, evaluation, and AI methods used in the included studies.

ChatGPT

This systematic review has several limitations that warrant acknowledgment. Firstly, our search was limited to three primary academic databases: Scopus, PubMed, and ACM Digital Library. While these sources are comprehensive, it's possible that relevant studies might exist in other databases or grey literature sources, potentially leading to a partial representation of the literature.

Secondly, we did not conduct further investigations into aspects with high or unclear risks from the bias assessment, such as patient selection, reference standard, and flow and timing. Given the prevalence of radiographic datasets, particularly publicly available CXRs, across most included studies, we assumed uniformity across these aspects. However, this assumption may overlook potential variations that could impact the overall assessment.

Lastly, due to the substantial heterogeneity in study designs, comparators, biomarker types, evaluation methodologies, and AI methods utilized in the included studies, we opted not to perform a meta-analysis. While meta-analysis could provide valuable insights, the diverse nature of the included studies made it challenging to ensure meaningful comparability across studies, potentially affecting the validity and reliability of the synthesized findings.

user

improve this:

Findings from this systematic review confirm the high potential of AI-based methods for TB detection. Among different kinds of AI, DL, or more specifically CNNs, is dominantly applied than other AI methods. It also has more stable and consistent results probably due to the high number of included studies that used radiographic data type. However, many studies used relatively small size datasets which may hinder the overall performance of DL methods. Lastly, although AI-based methods achieve high performances in TB detection tasks using various kinds of data types, most of them were experimented with in-domain datasets. Therefore, future studies should expand the experimental focus to include the domain-shift analysis which may be proven to be useful in representing real-world scenarios in the TB detection domain.

ChatGPT

The systematic review underscores the considerable promise of AI-based approaches in TB detection. Among the array of AI methodologies, Deep Learning (DL), particularly Convolutional Neural Networks (CNNs), emerges as the predominant choice. This preference for DL is attributed to its consistently robust performance, likely bolstered by the prevalence of studies employing radiographic data types.

However, a notable limitation across many studies is the utilization of relatively small datasets, which may constrain the efficacy of DL methods. Despite achieving commendable results, the potential of DL models could be further enhanced with larger and more diverse datasets.

Furthermore, while AI models demonstrate impressive performance on in-domain datasets, there's a notable gap in evaluating their robustness to domain shifts. Future research endeavors should prioritize conducting domain-shift analyses to better simulate real-world scenarios in TB detection. This approach would provide invaluable insights into the generalizability and applicability of AI-based methods beyond controlled settings.

user

improve this:

SH designed the systematic review following PRISMA 2020 Guidelines under the guidance of S-TL, BGC, and GBM. SH prepared and registered the protocol following the PRISMA-P 2015 Statement under GBM’s supervision. The search strategy was proposed by SH and refined by AA. All available records were downloaded by SH and stored in the UNSW OneDrive platform which could be accessed by all authors at any time. Screening and duplicate records deletion was performed by SH and IB by reading and assessing records’ titles, abstracts, and keywords. The screening results were reported to and confirmed by AA, S-TL, BGC, and GBM. Next, SH and AW performed the full-text assessment of passing records against the exclusion criteria. The results then were reported to and confirmed by AA, S-TL, BGC, and GBM. In the Included phase, SH and AA checked for studies included in the descriptive analysis. During the selection process (initial screening and full-text assessment), SH, IB, and AW tried to resolve disagreements between themselves first, while further opinions from AA and/or GBM were

sought if needed. All authors were informed and rechecked the results of each phase conducted during the systematic review. All authors read and agree on the final version of the manuscript.

ChatGPT

SH spearheaded the systematic review, adhering to the PRISMA 2020 Guidelines, with guidance from S-TL, BGC, and GBM. Under GBM's supervision, SH formulated and registered the protocol in line with the PRISMA-P 2015 Statement. The search strategy, initially proposed by SH, was refined in collaboration with AA. SH meticulously downloaded all available records, storing them on the UNSW OneDrive platform for accessibility to all authors.

During the screening process, SH and IB meticulously examined records' titles, abstracts, and keywords, with screening outcomes reviewed and confirmed by AA, S-TL, BGC, and GBM. Subsequently, SH and AW conducted thorough full-text assessments against exclusion criteria, with results reviewed and confirmed by the aforementioned authors. In the inclusion phase, SH and AA verified studies included in the descriptive analysis.

Throughout the selection process, any disagreements between SH, IB, and AW were first addressed internally, with additional input sought from AA and/or GBM if required. All authors were kept informed and reviewed the results of each phase during the systematic review. Ultimately, all authors collectively reviewed and approved the final manuscript version.

user

The first author is supported by the Department of Foreign Affairs and Trade (DFAT) Australia via the Australia Awards Scholarship (AAS).

ChatGPT

The first author acknowledges support from the Department of Foreign Affairs and Trade (DFAT) Australia through the Australia Awards Scholarship (AAS).
